# Identification of a contact zone and hybridization for two subspecies of the American pika (*Ochotona princeps*) within a single protected area

**DOI:** 10.1371/journal.pone.0199032

**Published:** 2018-07-11

**Authors:** Jessica A. Castillo Vardaro, Clinton W. Epps, Benjamin W. Frable, Chris Ray

**Affiliations:** 1 Department of Fisheries and Wildlife, Oregon State University, Corvallis, Oregon, United States of America; 2 Scripps Institution of Oceanography, University of California San Diego, La Jolla, California, United States of America; 3 Institute of Arctic and Alpine Research, University of Colorado-Boulder, Boulder, Colorado, United States of America; National Cheng Kung University, TAIWAN

## Abstract

Genetic variation is the basis upon which natural selection acts to yield evolutionary change. In a rapidly changing environment, increasing genetic variation should increase evolutionary potential, particularly for small, isolated populations. However, the introduction of new alleles, either through natural or human-mediated processes, may have unpredictable consequences such as outbreeding depression. In this study, we identified a contact zone and limited gene flow between historically separated genetic lineages of American pikas (*Ochotona princeps*), representing the northern and southern Rocky Mountain subspecies, within Rocky Mountain National Park. The limited spatial extent of gene flow observed may be the result of geographic barriers to dispersal, selection against hybrid individuals, or both. Our fine-scale population genetic analysis suggests gene flow is limited but not completely obstructed by extreme topography such as glacial valleys, as well as streams including the Colorado River. The discovery of two subspecies within this single protected area has implications for monitoring and management, particularly in the light of recent analyses suggesting that the pikas in this park are vulnerable to fragmentation and local extinction under future projected climates. Future research should focus on the fitness consequences of introgression among distinct genetic lineages in this location and elsewhere, as well as within the context of genetic rescue as a conservation and management strategy for a climate sensitive species.

## Introduction

Intraspecific genetic diversity is the most fundamental element of biodiversity and provides the basis for natural selection to yield evolutionary change [1–3]. When confronted with rapid environmental change, populations must either adapt *in situ* to new conditions, shift their distribution to more favorable environmental conditions, or face extinction [4]. Therefore, understanding patterns of genetic diversity and population structure is essential for developing and implementing effective conservation and management strategies for at risk populations [5, 6]. Local, regional, and historical processes all contribute to contemporary patterns of genetic diversity and population differentiation. Populations at the extremes of a species’ range, either geographic or climatic, may be most vulnerable to rapid, contemporary climate change. However, they may also represent reservoirs of adaptive potential if there is local adaptation to extreme environmental conditions [5–7].

Anthropogenic manipulation of genetic structure, whether intentional or accidental, also may influence adaptive potential. Translocations and augmentations, whereby individuals from one locality are moved to another location to found a new population or to supplement an existing population [8, 9] have been used to combat declining populations for decades. There are many well-known examples of mammalian reintroductions stemming from translocations, including wolves [10] and bighorn sheep [8, 11], as well as augmentations including, panthers [12] and bighorn sheep [8]. In addition to traditional attempts at demographic rescue by increasing population numbers, some more recent interventions have focused on genetic rescue which aims to increase population resilience by increasing overall genetic diversity and or targeting specific traits such as resistance to disease [12]. Best practices dictate that translocated individuals should come from closely-related genetic stock to avoid admixture among distinct evolutionary units that may have negative consequences such as maladaptation and outbreeding depression [11, 13]. Yet, some have raised the question of whether, in the face of extinction, more dramatic interventions such as interbreeding distantly related populations with different biogeographic history could prove beneficial [9]. For example, interbreeding with distantly related genetic lineages could introduce novel alleles or reintroduce ancestral alleles that were lost through drift, possibly having positive consequences for fitness [14]. Evolutionary rescue [15] and the potential genetic consequences of translocations [11, 16] are active and extensive areas of research. Nevertheless, these are difficult processes to study empirically outside a laboratory setting.

Naturally-occurring hybrid zones among distinct and formerly isolated genetic lineages provide opportunities to learn more about introgression, such as how genes spread through admixed populations and the subsequent effects on physiology and behavior [17, 18]. Among the well-studied natural hybrid zones, many are the result of secondary contact that occurred following the last ice age and thus occur in geographic clusters sometimes referred to as “suture zones” [17, 19–23]. Well-studied zones include mountain ranges in Europe and western North America [17, 19], and such taxa as grasshoppers in the French Alps [21] and gophers in the Rocky Mountains [24]. These studies shaped our early understanding of genetic introgression in hybrid zones. Barton and Hewitt [25, 26] argued that many hybrid zones are clines maintained by a balance between dispersal and selection against hybrids, which they refer to as “tension zones”. The characteristics of the cline (e.g., steepness and width) are therefore determined by the rate of gene flow, dispersal distance, and relative fitness of alleles between the two genetic sources [25, 26]. Recent developments in sequencing technologies and the inclusion of population genomic data into studies of hybrid zones has greatly increased our understanding of the evolutionary consequences of hybridization, but the increase in studies and associated data has also illuminated the complexity and variability of possible outcomes of hybridization [18].

**Fig 1:**
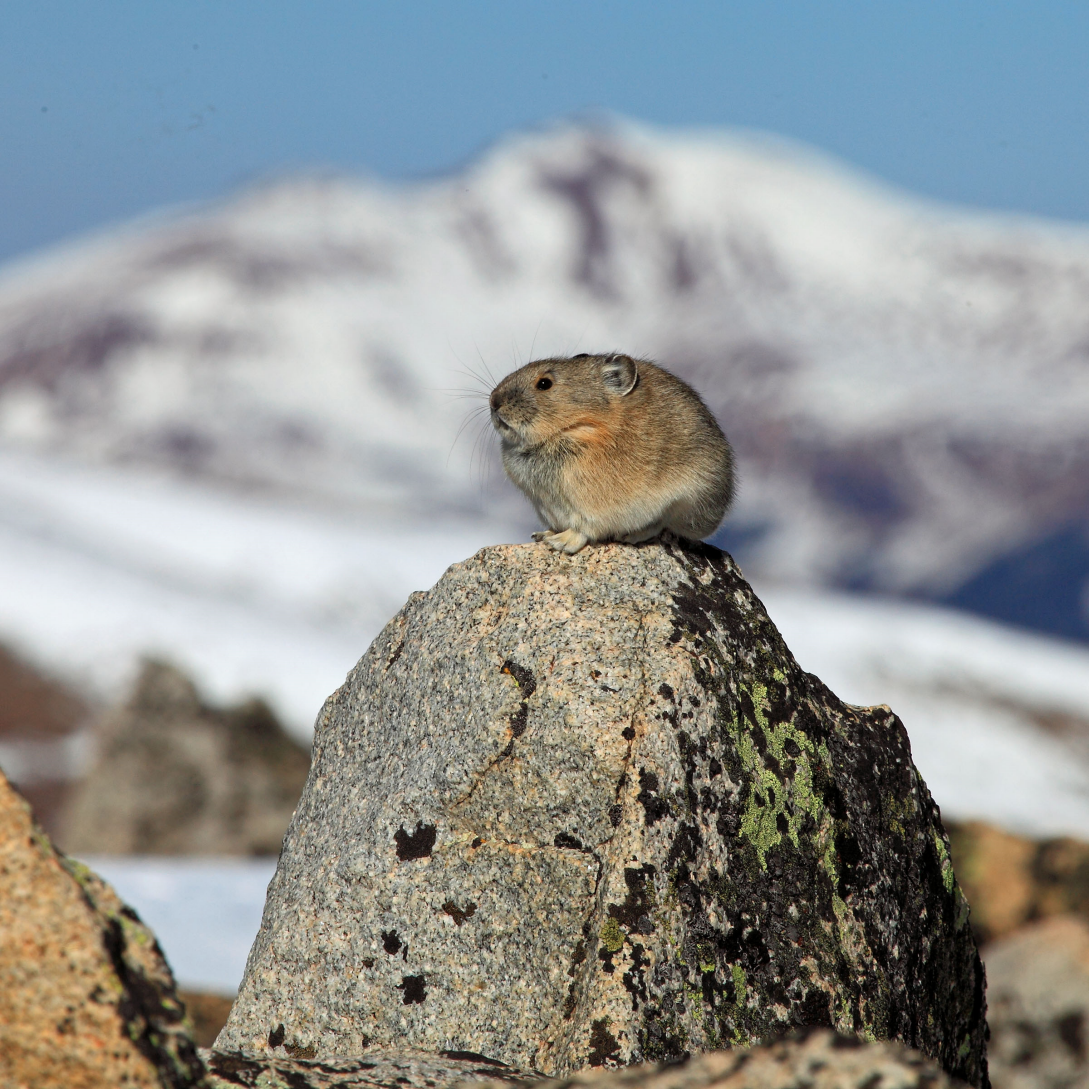
American pika (*Ochotona princeps*) in Rocky Mountain National Park, Colorado. Photo credit: Dick Orleans.

In this study, we describe a previously undocumented hybrid zone between two distinct genetic lineages of American pikas, *Ochotona princeps*, within Rocky Mountain National Park (ROMO) and discuss the implications for conservation of the species within this management unit and elsewhere. American pikas are often considered a sentinel species with respect to climate change due to numerous observations of local extinctions, particularly in lower, drier, and hotter habitats [27–30]. Recent extirpations at higher elevations among habitat thought to be more favorable to pikas have further worried biologists and managers [31]. Pikas are small lagomorphs (121–176 g, Fig 1) found throughout much of the intermountain western United States [32]. They are restricted to fractured rock habitats, such as talus slopes and lava flows, which provide refuge from predators and thermal buffering [32–36]. They cannot tolerate prolonged exposure to high temperatures and are therefore typically found at high elevations, but may persist at lower elevations and in hotter climates if there are suitable microclimatic refugia [37–40]. Predictive modeling has suggested widespread losses in the species’ distribution particularly in, but not limited to, low elevations [41]. More recently, models accounting for shifts in functional connectivity as well as climatic variables suggest a highly variable and idiosyncratic response to climate change [42]. Some populations are likely to persist, while others such as those in ROMO may be at high risk of extirpation [42]. American pikas are therefore considered a climate indicator species [43] and were petitioned to be listed under the Endangered Species Act in 2007. In 2010, the United States Fish and Wildlife Service (USFWS) concluded that listing of the species, or any portion of the species, was not currently warranted due to lack of scientific information [44]. Thus, understanding the factors that shape the distribution, genetic diversity, and adaptive potential for American pikas is of immediate conservation concern and underscores the need to explore innovative management strategies.

Previous work based on morphology, dialect, and both mtDNA and nuclear coding sequence data identified a potential historic contact zone between the northern and southern Rocky Mountain lineages in the vicinity of ROMO [45–47]. Establishing the existence of such a contact zone within a single protected area would inform management of the species within ROMO and, potentially, encourage investigation of such contact zones for other species. Furthermore, as conservationists are increasingly exploring genetic rescue as a management option [9, 48], natural hybrid zones present an opportunity to evaluate concerns around anthropogenic manipulation of genetic structure. Previous phylogeographic studies of American pikas utilized a combination of molecular markers that reflect historical gene flow [47, 49], whereas the current study investigates contemporary gene flow among historically separated populations using markers with mutation rates that are relatively moderate (mtDNA) and high (microsatellite loci) as compared to nuclear coding sequences. Here we present a fine-scale population genetic study evaluating this potential contemporary contact zone and evidence for gene flow between the Northern Rocky Mountain (*O*. *p*. *princeps*) and Southern Rocky Mountain (*O*. *p*. *saxatilis*) lineages [50]. Given the spatial distribution of behavioral evidence for a hybrid zone [45], the American pika’s philopatric behavior and low dispersal ability [32, 51, 52], as well as the potential for extreme topography and streams to limit dispersal [53], we expect any gene flow among the two lineages to be limited to locations separated by no more than a few kilometers and shaped by landscape features.

## Methods

### Study sites and genetic sampling

**Fig 2:**
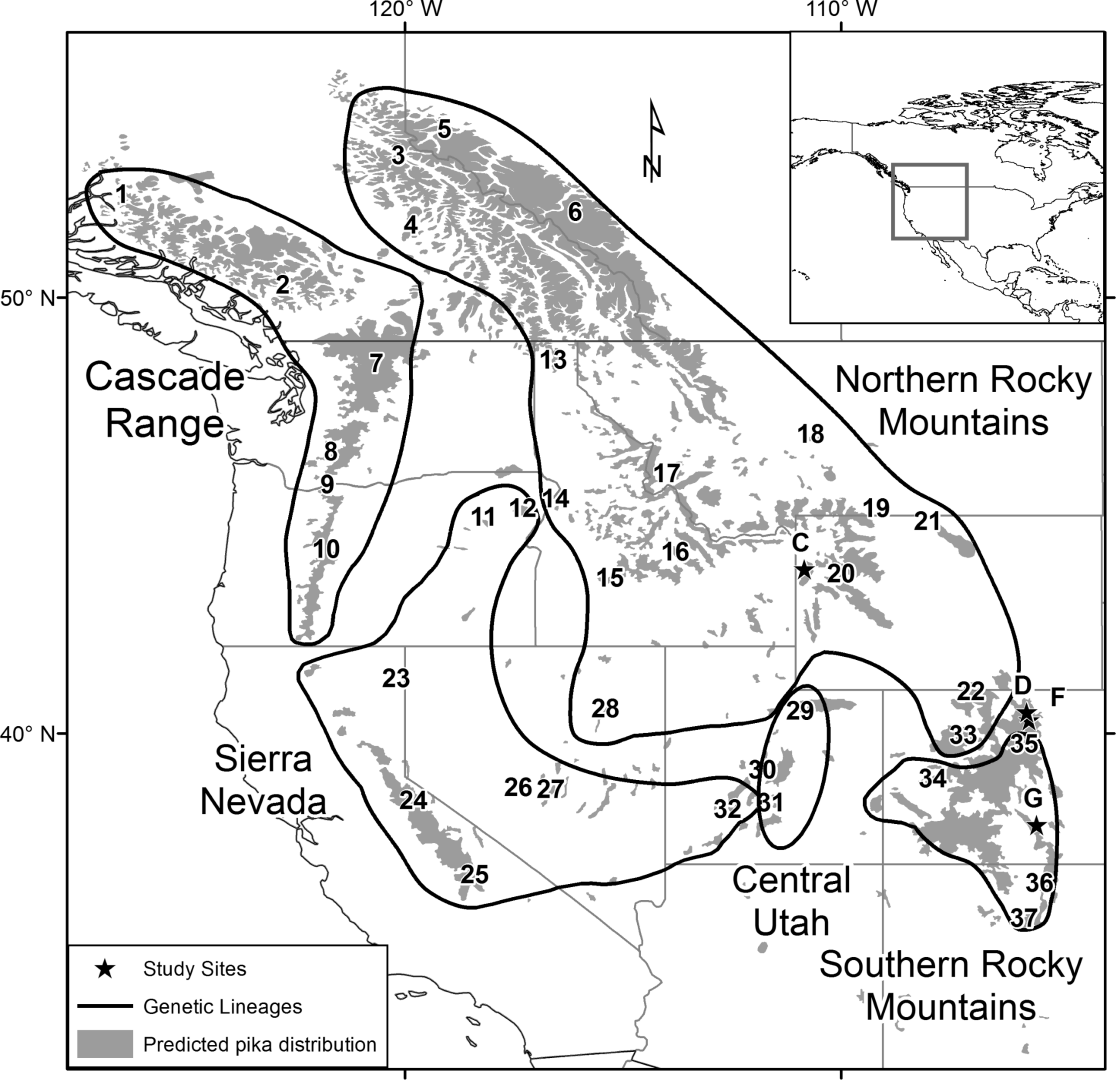
Map of major genetic lineages of American pikas and study sites. Study sites (numbered) and major mitochondrial lineages (black outlines) redrawn from Galbreath *et al*. 2010. Predicted distribution of American pikas (gray shading) derived from Kuchler potential natural vegetation [57, 58] according to Hafner and Sullivan [46]. Numbers refer to localities from Galbreath *et al*. 2010 and correspond to Table B in S1 File. Stars labeled with letters refer to sites from this study and correspond to Table B in S1 File. They are as follows: C) Grand Teton National Park, D) Rocky Mountain National Park north, F) Rocky Mountain National Park south, and G) Great Sand Dunes National Preserve.

In this study, we focus on ROMO as the context for contact between the two Rocky Mountain subspecies, in comparison with data from two additional study sites, Grand Teton National Park (GRTE) and Great Sand Dunes National Park (GRSA). GRTE and GRSA fall within the geographic range of the northern and southern Rocky Mountain lineages, respectively [47] (Fig 2). ROMO, however, does not fall within either subspecies range as defined in Galbreath et al. [47] (Fig 2). Detailed study design and microsatellite genotype data for these and other sites were previously reported for related studies [53]. Briefly, we collected fecal samples for genetic analyses between June 2010 and August 2014 through a combination of random, targeted, and opportunistic sampling. We collected random samples during standardized occupancy surveys conducted for other related studies [42, 54]. These survey locations were determined according to a generalized random stratified tessellation design [55] within potential pika habitat identified via remote sensing [54]. In addition to random sampling, we collected fecal samples opportunistically while traveling between survey locations, as well as through targeted searches of areas found to have pikas. We avoided collecting old fecal pellets by preferentially collecting pellets with green plant material inside to avoid degraded DNA. The color of the plant material fades from green to yellow within a few weeks to months [56]. We only collected distinct clusters of fecal pellets that were not contacting other previously deposited pellets to avoid contamination with DNA from other individuals. We collected fecal samples in paper coin envelopes and stored them dried until extraction. All field work was conducted as part of the Pikas in Peril? Project (PMIS #163377) and collections made under Scientific Research and Collection Permits (ROMO-2011-SCI-0032, GRSA-2010-SCI-0004, GRTE-2010-SCI-0079).

### Laboratory methods

We extracted genomic DNA from fecal samples using a modified AquaGenomic DNA extraction protocol (MultiTarget Pharmaceuticals LLC, Salt Lake City, UT, USA). We genotyped individuals at 24 microsatellite loci in four multiplex polymerase chain reactions (PCR) using a Qiagen Multiplex PCR kit (Qiagen, Valencia, CA, USA). Detailed PCR protocol, primer sequences, and methods for calling and screening microsatellite genotypes are provided in Castillo *et al*. [53]. In order to compare with previous phylogenetic analyses, we used the two primer pairs described in Galbreath *et al*. [49] to amplify the cytochrome-b oxidase (Cyt-b) and D-loop *mt*DNA region for a subset of our samples from ROMO, plus one from GRSA and GRTE. Due to the low quality and quantity of fecal DNA template as compared to fresh tissue, we designed an additional five primers to amplify smaller regions ranging from 483–566 bp (Table A in S1 File). All fragments were amplified in 10.5 μl reactions with final reagent concentrations of 2.25 mM MgCl_2_, 0.1 nM primers, 0.16 mM each dNTPs, 0.7 U *Taq* polymerase, and 0.5 μl template DNA. All reactions included a 15 minute (95°C) initial denaturation; 39 cycles of 30 sec. denaturation (95°C), 45 sec annealing (60°C), and 30 sec (72°C) extension; and a final 5 minute (72°C) extension. We sequenced all DNA fragments in both directions and visually inspected sequence alignments using Geneious 6.1.2 [59].

### Genetic structure within Rocky Mountain National Park

We performed a Bayesian clustering analysis in program Structure [60] to infer population structure within ROMO. We ran 10 replicates for each inferred number of populations (K = 1 to 10) totaling 100 replicates, with 100,000 MCMC steps each of burnin and run steps. We used eight regions within the national park, identified by geographic features, as sampling localities for location prior information in the admixture model, as well as correlated allele frequencies among populations as run parameters. We analyzed the model output using Structure Harvester [61] according to the ΔK method proposed by Evanno *et al*. [62]. We compared the optimal K from both ΔK and mean Ln Pr(X|K) methods to determine the best K value. We then used program Clumpp [63] to determine the optimal assignment of individuals across the ten runs for the best supported value of K. Finally, we visualized the output from Clumpp spatially using ArcMap 10.0 (ESRI, Redlands, California).

Given the limitations of Structure and methods to interpret such analyses, particularly in the case of two genetic clusters [64], we also performed a principal components analysis (PCA) and discriminant analysis of principal components (DAPC) [65] using the package “adegenet” [66] in R [67]. DAPC maximizes variation between groups while minimizing variation within groups and has the benefit of not relying on assumptions of Hardy-Weinberg proportions [65]. We performed the DAPC on all individual genotypes with no prior population assignment information by using the successive K-means approach, implemented by the *find*.*clusters* function, to identify the optimal number of groups based on Bayesian Information Criterion (BIC).

### Phylogenetic analysis

We obtained previously published sequence data covering the Cyt-b and D-loop region of the mitochondrial genome (c. 1650 bp) for 112 *Ochotona princeps* individuals, plus one *O*. *collaris*, from GenBank (Table B in S1 File). For Bayesian analysis, we selected the best data partitioning scheme and substitution models for each partition using a greedy algorithm in PartitionFinder 2.1.1 [68] with four a priori partitions for each codon of Cyt-b and one for D-Loop. We implemented the BIC corrected for small sample sizes to identify the best substitution model scheme (Table 1). We performed a Bayesian phylogenetic analysis of the partitioned matrix with MrBayes 3.1.2 [69] using the partitioning and models as detailed in Table 1. We performed two runs of four independent Markov chain Monte Carlo (MCMC) chains with 10M replicates each, sampling every 1000 generations or until the standard deviation of split frequencies between the two runs was less than 0.01. We discarded the first 25% of generations as burn-in for each run and then concatenated tree files. We found a maximum credibility tree (MCC), the tree with the highest product of posteriors for all nodes, using TreeAnnotator v1.8.2 [70]. We then edited the MCC tree in FigTree v.1.4.0 (http://tree.bio.ed.ac.uk/software/figtree/).

**Table 1:** Partitioning scheme and nucleotide substitution models used in Bayesian (MrBayes) phylogenetic analysis of two genes.

Partition	Substitution Model
CytB codon position 1	K80 + I
CytB codon position 2	HKY + I
CytB codon position 3	GTR + I + Γ
D-loop	HKY + I + Γ

### Population differentiation and genetic diversity

Once we identified two genetic clusters within ROMO (see results), we quantified genetic diversity and differentiation among the two groups, as well as GRTE and GRSA, from microsatellite genotypes. To measure deviation from panmixia assuming Hardy-Weingerg proportions, we calculated pairwise *F*_*ST*_ [71] using the “hierfstat” package [72] in R. Additionally, to measure population differentiation we calculated *D* [73, 74] using the “mmod” [75] package in R. We calculated geographic distance among study sites from the centroid of genotyped samples within each study site. We calculated expected heterozygosity (He) and allelic richness (Ar) corrected for sample size using the “hierfstat” package [72] in R. Finally, we calculated haplotype diversity (*h*) and nucleotide diversity (π) for the ROMO mtDNA dataset using DnaSP [76].

### Geographic cline analysis and hybrid detection

We fit the admixture proportion values from the Structure analysis for K = 2 to equilibrium cline models using the Metropolis-Hastings Markov chain Monte Carlo algorithm implemented in the R package HZAR [77]. We fit 15 candidate models that varied in the number of cline shape parameters estimated and selected the best model according to AIC corrected for small sample size. We were thus able to estimate the geographic center and width of the cline. Finally, we performed a hybrid detection analysis to estimate the probability that individual genotypes reflect genotype frequency categories corresponding to pure individuals, F1 or F2 hybrids, or backcrosses. We categorized individuals with *Q* > 0.99 in either northern or southern cluster as “pure” individuals and categorized all others as of unknown origin. We performed the hybrid detection analysis using the program newhybrids [78], implemented in the R package parallelnewhybrid [78], with 100,000 burnin reps and 500,000 sweeps. We performed ten replicate runs and averaged the posterior probabilities for each individual across all ten runs.

## Results

### Genetic data

After removing individual samples that either failed to amplify, were contaminated (contained more than 2 microsatellite peaks at any locus), or gave inconsistent genotypes, our final dataset included 230 genotyped individuals from ROMO, 194 from GRTE, and 54 from GRSA. After screening loci for significant deviations from expected Hardy Weinberg proportions and removing loci that failed to amplify consistently across sites, we included 22 microsatellite loci. Number of alleles per locus across all four sites ranged from 5 to 28 (mean = 14).

### Genetic structure

**Fig 3:**
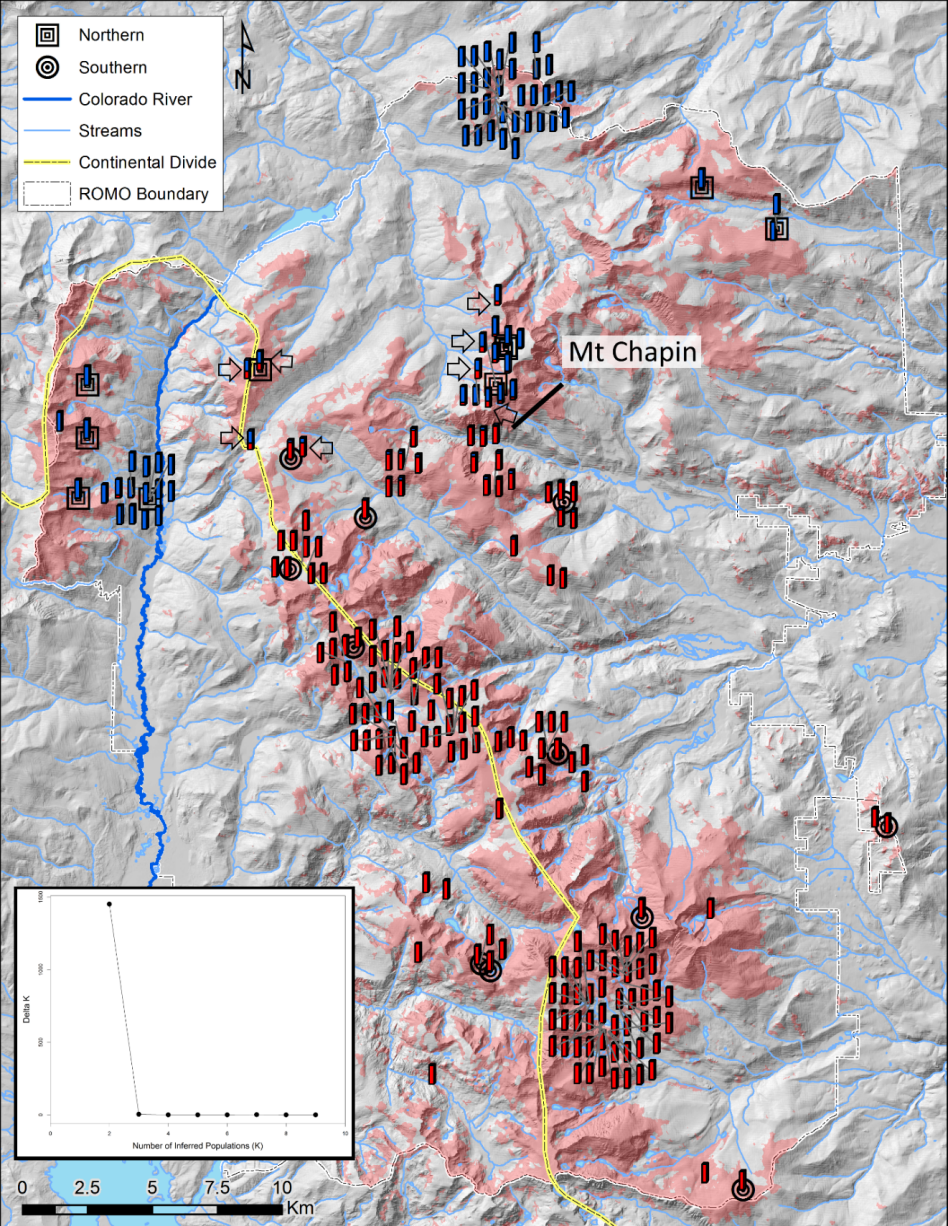
Population genetic structure within Rocky Mountain National Park. Individuals are shown as bar plots representing probability of assignment (q values) from the Structure analysis for K = 2. Individuals cluster geographically as a northern and southern population, separated by Mt. Chapin and the Colorado River. Concentric, black squares and circles indicate the placement of sequenced individuals into the northern and southern *mt*DNA lineages, respectively (Fig 6). Red shading indicates potential pika habitat [54]. Inset Figure shows ΔK values, indicating support for K = 2. Hillshade background was derived from the USGS National Elevation Dataset, streams and lakes were from the National Hydrography Dataset (https://nationalmap.gov).

**Fig 4:**
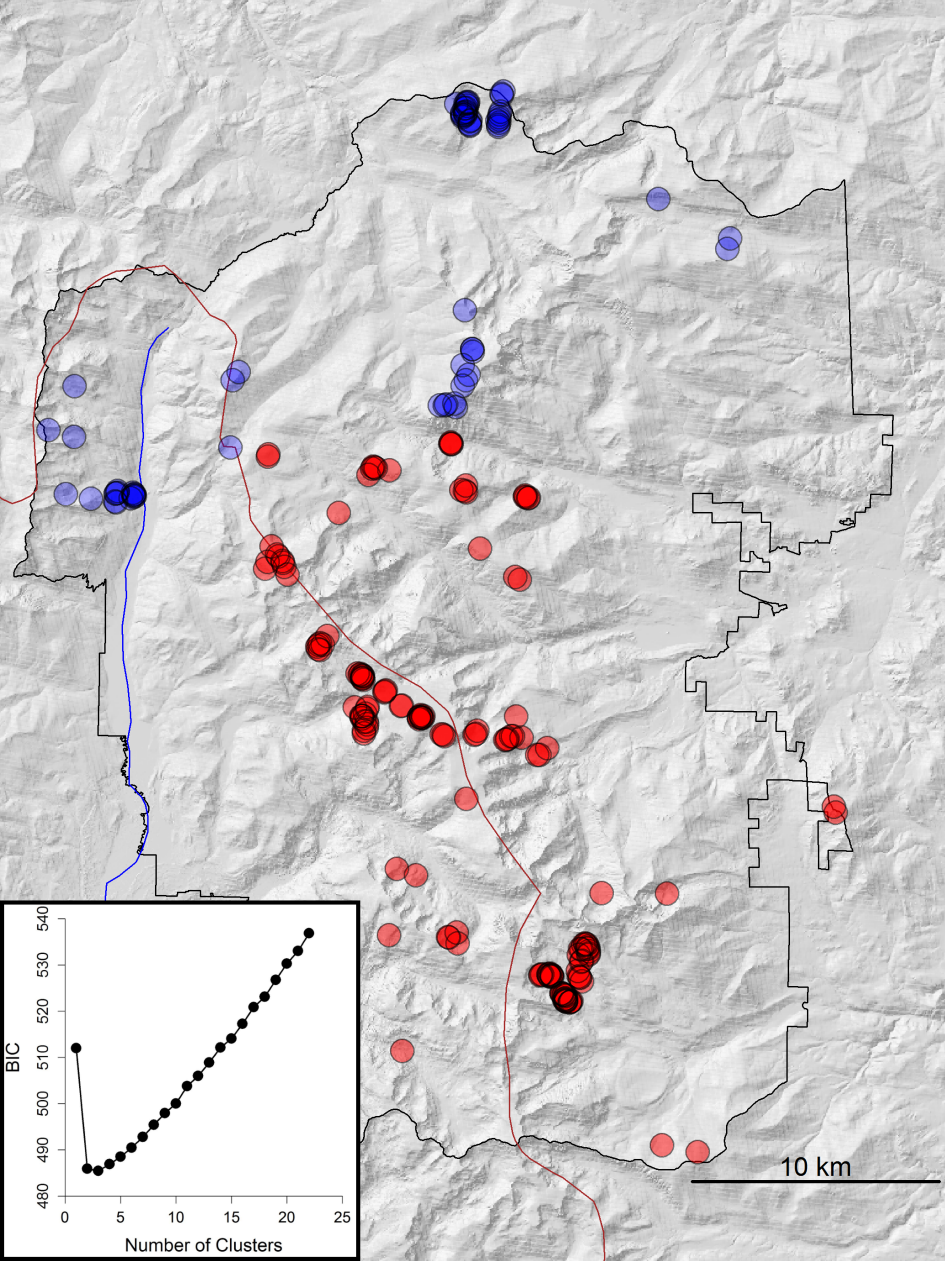
Results of the DAPC showing BIC support for two genetic clusters (inset) and assignment to either the north (blue) or south (red) clusters. Darker circles indicate multiple samples from that locality. Hillshade background was derived from the USGS National Elevation Dataset (https://nationalmap.gov).

**Fig 5:**
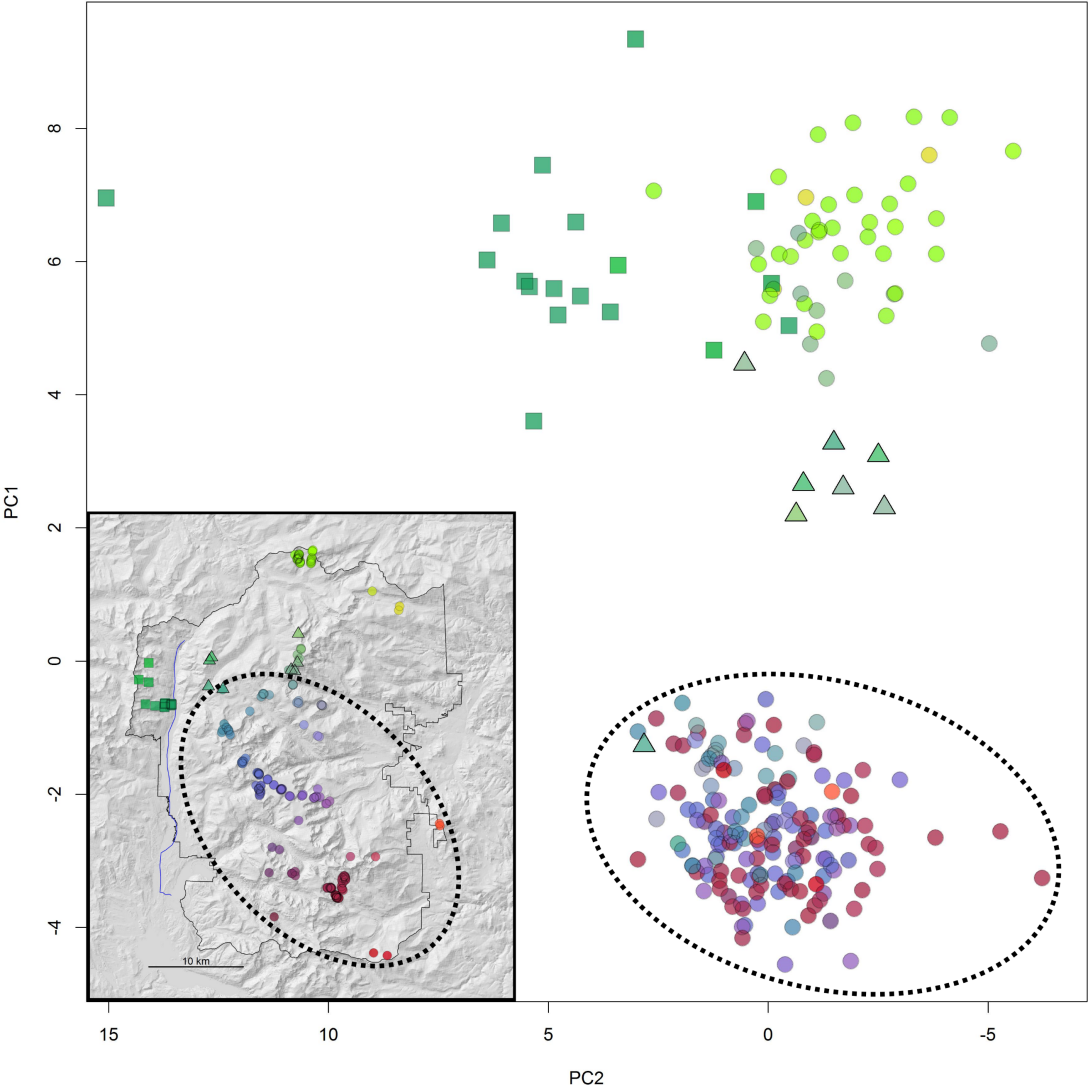
Principal component analysis of individual pika microsatellite genotypes. Points are color-coded according to geographic location and correspond to sampling locations in inset map of ROMO. Triangles represent individuals identified from the STRUCTURE analysis as having notably mixed ancestry (0.2 ≤ *Q* < 0.8). Points in the dashed oval correspond to sampling localities within the dashed oval in the inset map, which in turn correspond to red points in Fig 4. These points separate along PC1 and are geographically isolated by topography. The sampling localities west of the Colorado River (dark green squares west of blue line in inset) partially separate along PC2.

The Structure analysis supported two genetic clusters within ROMO (K = 2, Fig 3, Table A in S2 File and Figure A in S2 File). The DAPC likewise identified two clusters (Fig 4). All individuals were assigned to the same population clusters based on the DAPC (posterior membership probabilities > 0.99) and Structure analysis (*Q* ≥ 0.6, where *Q* is the proportion of the genome that originates from population K, also known as the admixture proportion [60]). Geographically, the two genetic clusters were roughly segregated into a northern and southern cluster. The northern cluster included individuals found north of Mt. Chapin or west of the Colorado River (Fig 3). The Structure analysis suggested some admixture occurred between the clusters (Fig 3). This was also supported by the PCA, where individuals identified as admixed in the Structure analysis (0.2 < *Q* < 0.8) had more intermediate values along the first principal component axis compared to individuals with higher *Q* values (Fig 5).

We subsequently performed two separate Structure analyses for the individuals assigned to the northern and southern clusters with *Q* ≥ 0.6. Within the northern group, there was support for three clusters, with one primarily in the northeast and another mostly restricted to the west of the Colorado River (Table B in S2 File and Figure B in S2 File). This was supported by the PCA, where individuals west of the Colorado River segregated along the 2^nd^ principal component axis (Fig 5). There was less support for population substructure within the southern group where there was some support for K = 2 and K = 6 (Table C in S2 File and Figure E in S2 File). Both results suggest isolation by distance with some restricted gene flow across streams and steep topography, but no major barriers to gene flow (Figure F in S2 File). Again, this was supported by a PCA of individuals within the southern cluster showing a latitudinal gradient along the first PCA (Figure G in S2 File).

### Phylogenetic analysis

**Fig 6:**
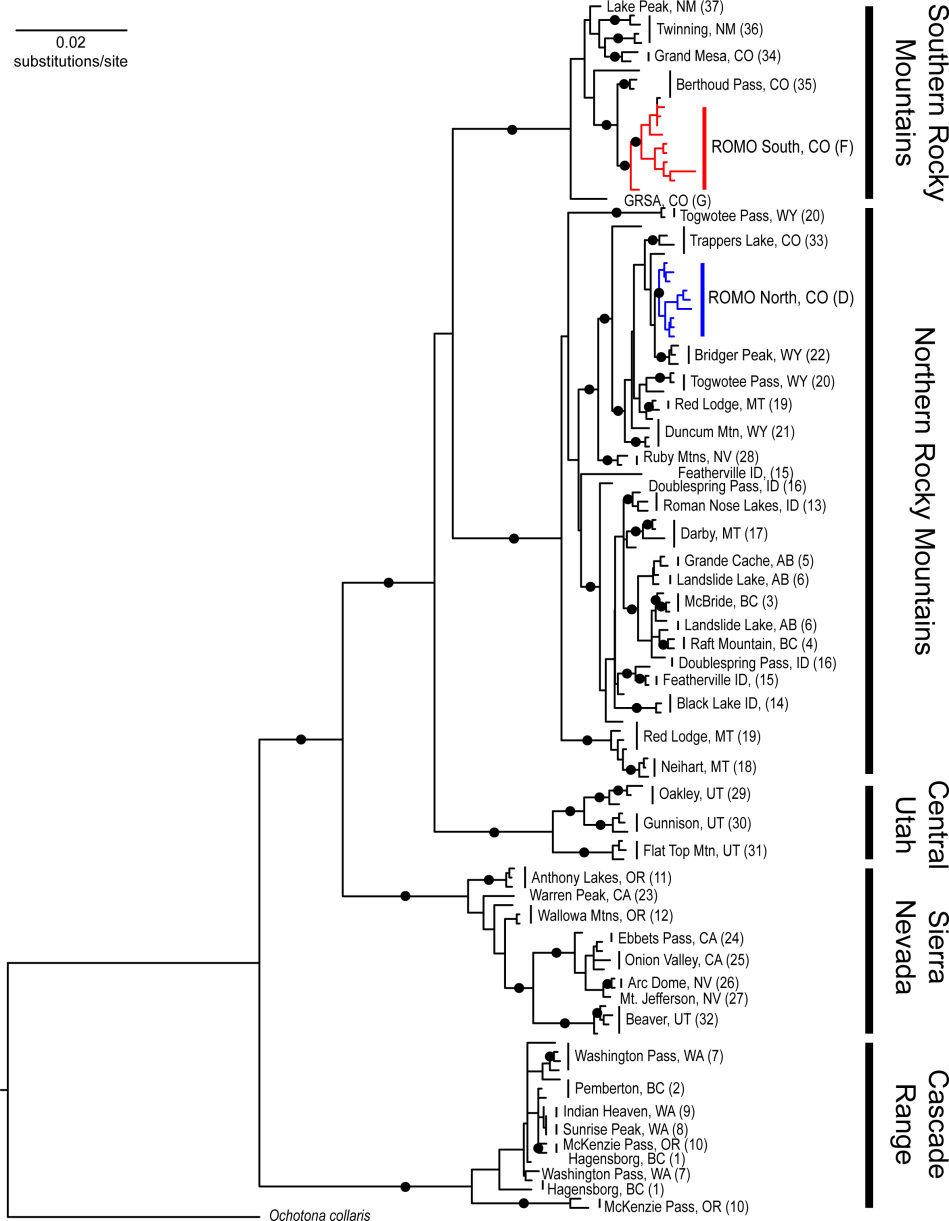
Maximum clade credibility phylogeny for *Ochotona princeps*. Black circles on branches indicate Bayesian posterior probabilities >95%. Samples from ROMO appear in both the northern (blue) and southern (red) Rocky Mountain lineages. Numbers and letters in parentheses refer to Fig 2 and are listed in Table B in S1 File.

We included 19 individuals from within ROMO and one from GRSA that resulted in good quality sequence data covering the 1650 bp target region, along with the 113 individuals from Galbreath *et al*. (2009) (Table B in S1 File). PartitionFinder supported the a priori scheme with substitution models summarized in Table 1. The MrBayes runs achieved convergences within the first 10M generations. We recovered a MCC phylogeny with strong support (posterior probability > 0.9) for each of the five previously identified clades of *O*. *princeps* (Fig 6). Among the samples from ROMO, 9 individuals grouped within the northern Rocky Mountain lineage, *O*. *p*. *princeps*, and 10 individuals were grouped within the southern Rocky Mountain lineage, *O*. *p*. *saxatilis* (Fig 6). The results were consistent with both the Structure and DAPC analyses based on microsatellite genotypes (Fig 3).

### Population differentiation and genetic diversity

**Fig 7:**
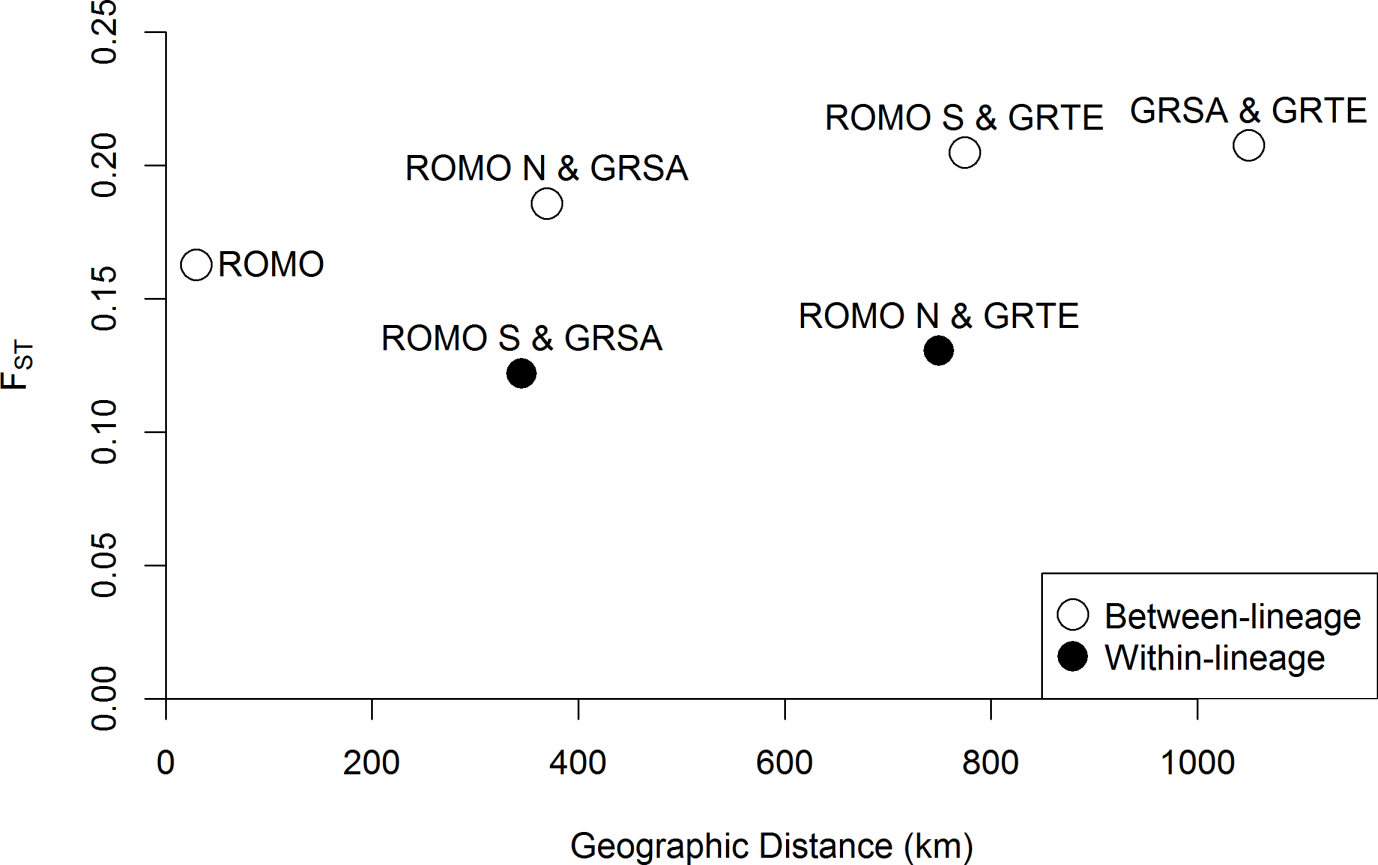
Pairwise *F*_*ST*_ between study sites plotted against geographic distance. Pairs of sites within the same genetic lineage (filled circles) and between genetic lineages (open circles).

Pairwise *F*_*ST*_ calculated from microsatellite genotypes was greater between lineages than within lineages (Table 2) and increased with geographic distance (Fig 7). *F*_*ST*_ was greater between ROMO N and ROMO S, at a distance of <20 km, than within-lineages estimates hundreds of km apart, but less than between-lineage comparisons involving GRTE or GRSA. Pairwise *D* followed the same pattern (Table 2). Microsatellite allelic richness was greater within ROMO than either GRTE or GRSA, but these differences were not significant (-1.23 < *t* 1.38, p > 0.1). Likewise, microsatellite heterozygosity was similar among the study sites (Table 3) and relatively high compared to 9 other sites from a previous study [79]. Haplotype (*h*) and nucleotide diversity (π) calculated from mtDNA sequences was similar between ROMO N and ROMO S. We identified 7 haplotypes in each site. Genetic diversity was slightly higher in ROMO N (*h* = 0.00199, π = 0.92) than ROMO S (*h* = 0.00163, π = 0.91).

**Table 2:** Genetic differentiation among study sites for multilocus microsatellite genotypes.

	GRSA	GRTE	ROMO N	ROMO S
GRSA	-	0.63	0.56	0.38
GRTE	0.21	-	0.33	0.62
ROMO N	0.19	0.13	-	0.48
ROMO S	0.12	0.20	0.16	-

Pairwise population D (Jost 2008, above diagonal) and Fst (below diagonal) for 22 microsatellite loci, between Rocky Mountain National Park north (ROMO N) and south (ROMO S), Grand Teton National Park (GRTE), and Great Sand Dunes National Park (GRSA). Shaded cells indicate comparisons within major genetic lineages.

**Table 3:** Sample size for microsatellite genotypes (n), allelic richness (Ar), observed heterozygosity (Ho) and expected heterozygosity (Hs) for each study site.

Site	n	Ar	Ho	Hs
GRSA	54	7.23	0.62	0.73
GRTE	194	7.31	0.64	0.69
ROMO N	69	7.88	0.60	0.69
ROMO S	161	8.03	0.58	0.73

### Geographic cline analysis and hybrid detection

The cline width for the best supported model was approximately 8.5 km with a two log-likelihood support range of 6.5–11.25 km (Table E in S2 File and Figure H in S2 File). The cline width estimates from the top 13 models were also within this range (Table E in S2 File). The estimated cline center was approximately 27.75 km north of the southernmost sampled individual, just south of Mt. Chapin (Table E in S2 File and Figure I in S2 File). Admixed individuals from the geographic north were characterized as either F2 hybrids or north backcrosses with posterior probability ≥ 0.6 (Figure J in S2 File). The one admixed individual from the geographic south was characterized as a south backcross with posterior probability 0.93. There was some support for backcrosses within the individuals with admixture proportions *Q* ≥ 0.8 (Figure J in S2 File).

## Discussion

We identified a previously undescribed contact zone between the northern and southern Rocky Mountain lineages within Rocky Mountain National Park. Our results were consistent across all three different types of analyses (STRUCTURE, DAPC, and phylogenetic) and therefore robust to the K = 2 conundrum common in STRUCTURE analyses [64]. Moreover, we determined there was contemporary gene flow between the two lineages. Galbreath *et al*. [47] identified shared alleles among northern and southern Rocky Mountain populations in sequences of two nuclear introns (protein kinase C iota and mast cell growth factor) and determined that this was the result of gene flow since the last glacial maximum when receding montane glaciers were no longer barriers to dispersal. However, based on more-rapidly mutating mtDNA loci, they determined that the Colorado River represents a relatively recent barrier preventing contemporary gene flow between the two lineages [47]. In their taxonomic revision, Hafner and Smith [50] described the northern and southern subspecies’ ranges as occurring on either side of the Colorado River. The microsatellite markers used in this study have a considerably higher mutation rate than mtDNA, reflecting evolutionary processes within a few tens of generations [80–82], as compared to many hundreds to thousands of generations for mtDNA [82]. We determined that the Colorado River is not an adequate delineation of the geographic boundary between the two subspecies (Figs 2–4 and S2 File). However, the river does appear to be at least a partial barrier to gene flow (Fig 5 and S2 File).

Our results indicate contemporary gene flow between the two subspecies within ROMO, evidenced by intermediate levels of population differentiation as compared to estimates between populations from different or the same genetic lineage (Table 2), as well as the PCA and Structure analyses. However, admixture appears geographically limited to within a <10 km zone (Fig 3, Table E in S2 File, Figures H and I in S2 File). The geographic cline analysis should be interpreted with caution because 1) the area within and immediately surrounding the inferred hybrid zone was not exhaustively sampled, and 2) the analysis assumes a linear cline with minimal variation perpendicular to the cline [77]. We observed genetic structure on either side of the Colorado River (Figure C in S2 File), therefore this analysis should be repeated in the future with more extensive sampling and possibly omitting those individuals west of the Colorado River. Nevertheless, the results of the analysis were consistent with low dispersal ability further limited by geographic features observed in ROMO and other study areas [53]. Gaps in pika habitat, the Colorado River, and glacial valleys appear to contribute to genetic structure within (Figures C and E in S2 File) and among genetic lineages (Figs 2 and 3) despite their close geographic proximity. Alternately, the observed restricted gene flow among genetic lineages may be the result of only relatively recent contact, or possibly selection against hybrid individuals. This last hypothesis in particular warrants further investigation.

We did not find individuals with notably mixed ancestry in the southern cluster, with the exception of a single, likely backcrossed individual, collected just east of the continental divide (Fig 5). This pattern may be the result of more frequent dispersal of individuals from south to north. Previous research identified hybrid vocalizations at the headwaters of the Colorado River along the continental divide (Somers 1973), <5 kilometers from where we identified admixture among individuals. That study suggested that vocalizations were indicative of ancestry rather than learned behavior; and a subsequent study revealed that hybrid individuals reared in captivity do in fact produce hybrid vocalizations (Somers, personal communication). Additional observations of hybrid vocalizations outside ROMO suggest that further investigation should be made into the geographic extent and degree of admixture between these two genetic lineages. Dispersal is not sex-biased in pikas [83, 84], therefore differences in observed patterns between mtDNA and microsatellite analyses most likely do not reflect differences in dispersal between males and females. However, the sex of both parents and offspring may affect hybrid survival and fitness if for example traits are sex-linked or play a role in mate choice, such as in chemosensory behavior or vocalizations [14, 85–87]. Future research should investigate whether there is directional gene flow and if so, is it the result of dispersal patterns or selection for or against particular hybrid combinations.

Recent research has shown variation in population persistence across the species range over the past century [36, 88], as well variation in predicted future trends for American pikas that do not necessarily conform to a “colder is always better” scenario [36, 42, 54]. This may be in part driven by fine-scale microhabitat characteristics or other factors that are not captured by most models. Local adaptation and past biogeographic history likely also play important roles in population persistence such that populations that have experienced hotter climates in the past may be better prepared to deal with a warming climate in the future. We therefore might expect different responses to climate change between populations of the northern and southern Rocky Mountain lineages currently living under similar environmental conditions. What does this mean for hybrid lineages? Our research suggests, based on higher allelic richness, that gene flow between ROMO N and ROMO S has potentially increased genetic diversity within ROMO. In general, higher genetic diversity is thought to increase evolutionary potential in the face of rapid, environmental change [89]. However, gene flow may counteract local adaptation [90, 91]. Possible future scenarios for ROMO under a changing climate include: 1) resilience as a result of greater genetic diversity increasing adaptive potential through novel gene combinations, 2) decreased resilience as a result of the spread of maladapted genes from one lineage to the other, 3) increased resilience as a result of the spread of adaptive genes from one lineage to the other, or 4) little or no effect of admixture on resilience. Evidence from translocated bighorn sheep suggest adaptation to local environmental conditions was a strong determinant of translocation success in some cases [92], while other examples suggest high phenotypic plasticity and translocation success even within populations including subspecies hybrids [11]. Our study relied on neutral genetic markers to describe underlying genetic processes, therefore to address these hypotheses future research should seek to identify potential adaptive variation among these and other pika populations. Such studies could inform the feasibility of management actions such as translocating pikas within ROMO or among populations separated by greater geographic and environmental conditions, as has been proposed [93, 94].

The presence of two distinct genetic lineages as well a hybrid zone within a single park boundary presents some interesting, and potentially complicated, implications for management of this species within ROMO. Previous research demonstrated that there are five major American pika genetic lineages, each with independent evolutionary trajectories [49]. Galbreath et al. further suggest that these lineages should be considered distinct evolutionarily significant units [95] for management purposes. The 2010 decision by the USFWS to not list the American pika, or any subpopulations, under the ESA was based largely on subspecies revisions [50] according to these five independent lineages [44]. Should certain subspecies be listed in the future, pikas in ROMO could, in theory, be subject to different federal regulations, despite coexisting within a single management unit. One recent study suggested that pika in ROMO may be at particularly high risk of extirpation [42], but did not consider those subspecies separately. To further complicate this scenario, there are not clear guidelines under the ESA for treatment of hybrid individuals [96–98]. One example of successful translocations among subspecies and protection of hybrid offspring under the ESA is the introduction of Texas panthers (*Puma concolor stanleyana*) to populations of Florida panthers (*P*. *c*. *coryi*) [12]. In contrast, Allendorf et al. [99] recommended only pure Westslope cutthroat trout (*Oncorhynchus clarki lewisi*) be protected under the ESA and not hybrids with Yellowstone cutthroat trout (*O*. *c*. *bouvieri*) or rainbow trout (*O*. *mykiss*). While the ESA is a powerful management tool, it is often challenging to reconcile complex ecological and evolutionary processes with structured legal decisions.

## Supporting information

S1 File Mitochondrial DNA primer and sequence details. (DOCX)

S2 File Results from population genetic Structure, hzar, and newhybrids analysis of American pikas within Rocky Mountain National Park, based on 22 microsatellite loci. (DOCX)

S3 File Microsatellite genotypes and sampling localities. (CSV)
