# Supplementary material for: Identification of a contact zone and hybridization for two subspecies of the American pika (Ochotona princeps) within a single protected area
Source: PLoS One. 2018 Jul 11;13(7):e0199032. doi: 10.1371/journal.pone.0199032 (PMC6040701; doi:10.1371/journal.pone.0199032)
Supplement: S1 File — (DOCX) [file pone.0199032.s001.docx]

Identification of a contact zone and hybridization for two subspecies of American pikas (*Ochotona princeps*) within a single protected area

Authors: Jessica A. Castillo Vardaro^*^, Clinton W. Epps, Benjamin W. Frable, Chris Ray

*Corresponding author

Email: [Castillo.Jessica.A@gmail.com](mailto:Castillo.Jessica.A@gmail.com) (JACV)

**Table A.** Primer pairs, fragment lengths, and sequences for additional primers, used to amplify cytochrome *b* and D-loop from fecal DNA.

| Forward Primer | Reverse Primer | Length (bp) |
| --- | --- | --- |
| PIKA01: Galbreath *et al.* 2009 | PIKA02: Galbreath *et al.* 2009 | 797 |
| PIKA01: Galbreath *et al.* 2009 | PIKA_cytB_R2:  ACGGTTGCYCCTCAGAARGA | 483 |
| PIKA_cytB_F2:  GGYGCATCCATATTCTTCAT | PIKA02: Galbreath *et al.* 2009 | 493 |
| PIKA03: Galbreath *et al.* 2009 | PIKA_Dloop_R4:  TGTCCTAGGAAGRRTTGCAC | 566 |
| PIKA_Dloop_F3:  CTGCAGCYTRATCGAGAACA | PIKA_Dloop_R3:  TGCTGGTTTCACGGAGGATG | 555 |

**Table B.** Sample IDs, Genbank accession numbers, and sampling localities. Locality IDs refer to Figure s 1, 3, and 4.

| Sample Name | Genbank ID | Locality | Locality ID | Published in |
| --- | --- | --- | --- | --- |
| CUMV20735, 20741, 20742 | EU591048, EU591049 | Hagensborg BC | 1 | Galbreath *et al.* 2009 |
| CUMV20746, 20585, 20584 | EU591052, EU591054, EU591055 | Pemberton BC | 2 | Galbreath *et al.* 2009 |
| CUMV20715-20717 | EU591038-EU591040 | McBride BC | 3 | Galbreath *et al.* 2009 |
| CUMV20770, 20772 | EU591058, EU591060 | Raft Mountain BC | 4 | Galbreath *et al.* 2009 |
| CUMV20589, 20724 | EU591042, EU591045 | Grande Cache AB | 5 | Galbreath *et al.* 2009 |
| CUMV20591, 20696, 20698, 20699 | EU591031, EU591032, EU591034, EU591035 | Landslide Lake AB | 6 | Galbreath *et al.* 2009 |
| CUMV20284, 20287, 20288, 20293, 20295 | EU590991-EU590995 | Washington Pass WA | 7 | Galbreath *et al.* 2009 |
| CUMV20302, 20304 | EU590997, EU590999 | Sunrise Peak WA | 8 | Galbreath *et al.* 2009 |
| CUMV20307, 20310 | EU591000, EU591001 | Indian Heaven WA | 9 | Galbreath *et al.* 2009 |
| CUMV21152, 20630-20632 | EU591061, EU591063-EU591065 | McKenzie Pass OR | 10 | Galbreath *et al.* 2009 |
| CUMV20637, 20642, 20576 | EU591066, EU591069, EU591070 | Anthony Lakes OR | 11 | Galbreath *et al.* 2009 |
| CUMV20575, 20574, 20649 | EU591072, EU591074, EU591075 | Wallowa Mtns OR | 12 | Galbreath *et al.* 2009 |
| CUMV20062, 20278, 20282 | EU590987-EU590990 | Roman Nose Lakes ID | 13 | Galbreath *et al.* 2009 |
| CUMV20260, 20262, 20263, 20268 | EU590982-EU590985 | Black Lake ID | 14 | Galbreath *et al.* 2009 |
| CUMV20248, 20251, 20254 | EU590976, EU590978, EU590980 | Featherville ID | 15 | Galbreath *et al.* 2009 |
| CUMV20244, 20246, 20247 | EU590973-EU590975 | Doublespring Pass ID | 16 | Galbreath *et al.* 2009 |
| CUMV20237-20240 | EU590967-EU590970 | Darby MT | 17 | Galbreath *et al.* 2009 |
| CUMV20211, 20214, 20217 | EU590958, EU590959, EU590960 | Neihart MT | 18 | Galbreath *et al.* 2009 |
| CUMV20223, 20224, 20226, 20230, 20236 | EU590962-EU590966 | Red Lodge MT | 19 | Galbreath *et al.* 2009 |
| CUMV20180, 20181, 20186, 20189, 20190 | EU590946-EU590950 | Togwotee Pass WY | 20 | Galbreath *et al.* 2009 |
| CUMV20194, 20199, 20201, 20208 | EU590951, EU590953-EU590955 | Duncum Mtn WY | 21 | Galbreath *et al.* 2009 |
| CUMV20165, 20169, 20177 | EU590940, EU590943, EU590945 | Bridger Peak WY | 22 | Galbreath *et al.* 2009 |
| CUMV20682 | EU591020 | Warren Peak CA | 23 | Galbreath *et al.* 2009 |
| CUMV20683, 20684 | EU591022, EU591023 | Ebbets Pass CA | 24 | Galbreath *et al.* 2009 |
| CUMV20593, 20686, 20687 | EU591025-EU591027 | Onion Valley CA | 25 | Galbreath *et al.* 2009 |
| CUMV20312, 20315 | EU591002, EU591005 | Arc Dome NV | 26 | Galbreath *et al.* 2009 |
| CUMV20322 | EU591012 | Mt Jefferson NV | 27 | Galbreath *et al.* 2009 |
| CUMV20689, 20692 | EU591029, EU591030 | Ruby Mtns NV | 28 | Galbreath *et al.* 2009 |
| CUMV20573, 20572, 20658 | EU591076, EU591079, EU591080 | Oakley UT | 29 | Galbreath *et al.* 2009 |
| Sample Name | Genbank ID | Locality | Locality ID | Published in |
| CUMV20678, 20571, 20660 | EU591081, EU591082, EU591085 | Gunnison UT | 30 | Galbreath *et al.* 2009 |
| CUMV20569, 20663, 20669 | EU591086, EU591087, EU591090 | Flat Top Mtn UT | 31 | Galbreath *et al.* 2009 |
| CUMV20671, 21156, 21157, 20677 | EU591091-EU591094 | Beaver UT | 32 | Galbreath *et al.* 2009 |
| CUMV20154, 20156-20158 | EU590935, EU590937-EU590939 | Trappers Lake CO | 33 | Galbreath *et al.* 2009 |
| CUMV20149, 20152 | EU590933, EU590934 | Grand Mesa CO | 34 | Galbreath *et al.* 2009 |
| CUMV20127, 20128, 20131, 20137 | EU590926-EU590929 | Berthoud Pass CO | 35 | Galbreath *et al.* 2009 |
| CUMV20115, 20116, 20121, 20124 | EU590921-EU590924 | Twining NM | 36 | Galbreath *et al.* 2009 |
| CUMV20335 | EU591017 | Lake Peak NM | 37 | Galbreath *et al.* 2009 |
| GRSA_001 | MG700611 | GRSA | G | This study |
| ROMO_008 | MG700609 | ROMO | F | This study |
| ROMO_015 | MG700607 | ROMO | F | This study |
| ROMO_018 | MG700604 | ROMO | D | This study |
| ROMO_019 | MG700603 | ROMO | D | This study |
| ROMO_023 | MG700610 | ROMO | F | This study |
| ROMO_026 | MG700605 | ROMO | F | This study |
| ROMO_036 | MG700596 | ROMO | D | This study |
| ROMO_037 | MG700612 | ROMO | D | This study |
| ROMO_042 | MG700608 | ROMO | D | This study |
| ROMO_048 | MG700606 | ROMO | F | This study |
| ROMO_053 | MG700594 | ROMO | D | This study |
| ROMO_062 | MG700598 | ROMO | D | This study |
| ROMO_069 | MG700597 | ROMO | D | This study |
| ROMO_089 | MG700602 | ROMO | F | This study |
| ROMO_208 | MG700613 | ROMO | D | This study |
| ROMO_353 | MG700600 | ROMO | F | This study |
| ROMO_380 | MG700601 | ROMO | F | This study |
| ROMO_383 | MG700599 | ROMO | F | This study |
| ROMO_394 | MG700595 | ROMO | F | This study |
| O. collaris | AF348080 |  |  | Lin *et al.* 2002 |
