# Supplementary material for: Identification of a contact zone and hybridization for two subspecies of the American pika (Ochotona princeps) within a single protected area
Source: PLoS One. 2018 Jul 11;13(7):e0199032. doi: 10.1371/journal.pone.0199032 (PMC6040701; doi:10.1371/journal.pone.0199032)
Supplement: S2 File — (DOCX) [file pone.0199032.s002.docx]

Identification of a contact zone and hybridization for two subspecies of American pikas (*Ochotona princeps*) within a single protected area

Authors: Jessica A. Castillo Vardaro^*^, Clinton W. Epps, Benjamin W. Frable, Chris Ray

*Corresponding author

Email: [Castillo.Jessica.A@gmail.com](mailto:Castillo.Jessica.A@gmail.com) (JACV)

**Table A.** Summary of each of ten Structure runs for K = 1 to 10 for all genotyped pikas.

| K | Reps | Mean LnP(K) | Stdev LnP(K) | Ln'(K) | \|Ln''(K)\| | Delta K |
| --- | --- | --- | --- | --- | --- | --- |
| 1 | 10 | -17866.86 | 0.804 |  |  |  |
| 2 | 10 | -15889.35 | 1.289 | 1977.510 | 1869.740 | 1450.957 |
| 3 | 10 | -15781.58 | 11.361 | 107.770 | 50.140 | 4.413 |
| 4 | 10 | -15723.95 | 58.053 | 57.630 | 0.730 | 0.013 |
| 5 | 10 | -15665.59 | 64.704 | 58.360 | 15.300 | 0.236 |
| 6 | 10 | -15622.53 | 75.953 | 43.060 | 25.260 | 0.333 |
| 7 | 10 | -15554.21 | 75.291 | 68.320 | 102.620 | 1.363 |
| 8 | 10 | -15588.51 | 175.414 | -34.300 | 50.840 | 0.290 |
| 9 | 10 | -15571.97 | 192.768 | 16.540 | 40.220 | 0.209 |
| 10 | 10 | -15595.65 | 168.342 | -23.680 |  |  |


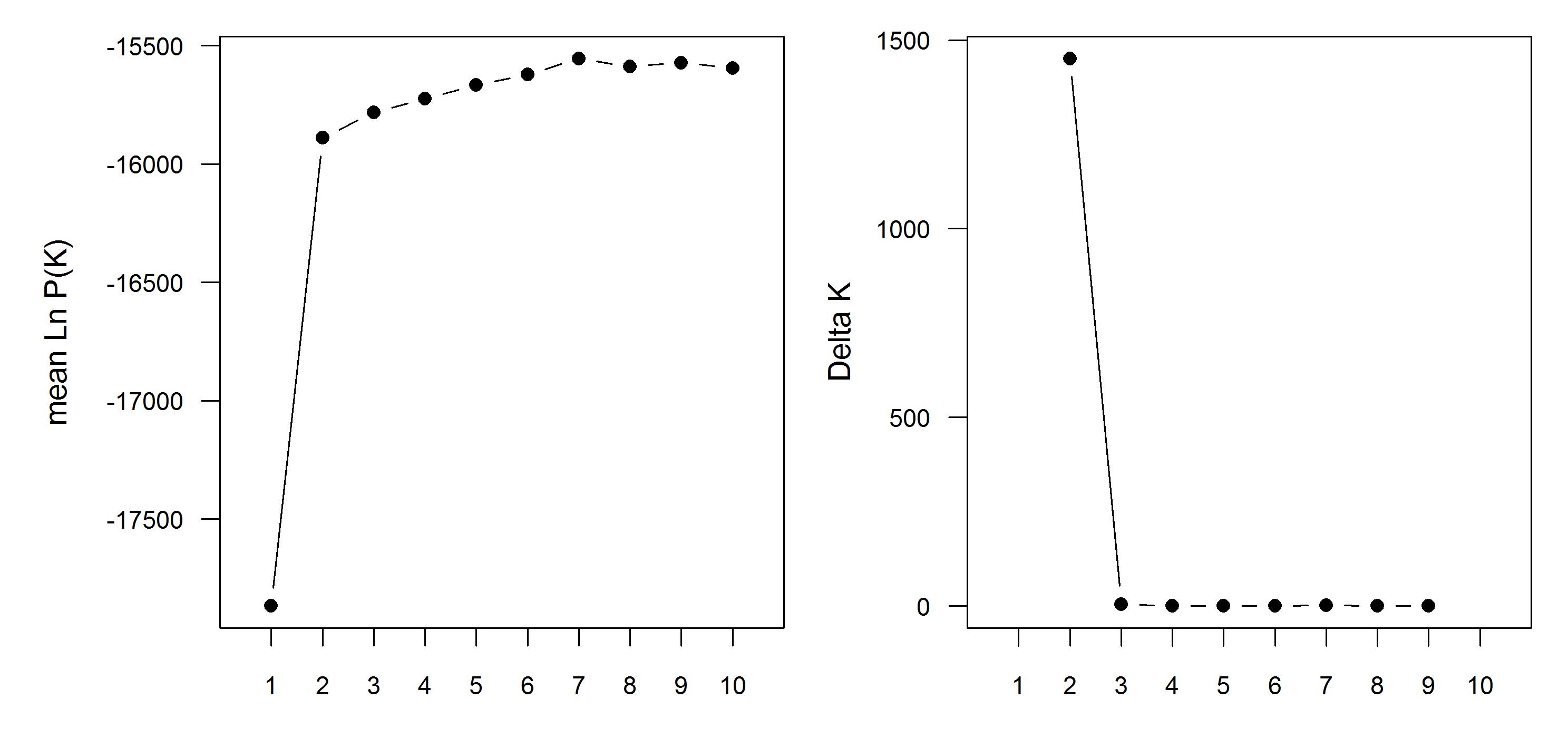


**Figure A.** Plot of mean Ln P(K) and Delta K showing support for K = 2 for all genotyped pikas.

**Table B** Summary of each of ten Structure runs for K = 1 to 10 for the subset of pika genotypes assigned to the northern cluster with admixture proportion *Q* ≥ 0.6 in the first Structure analysis.

| K | Reps | Mean LnP(K) | Stdev LnP(K) | Ln'(K) | \|Ln''(K)\| | Delta K |
| --- | --- | --- | --- | --- | --- | --- |
| 1 | 10 | -4522.07 | 1.6958 |  |  |  |
| 2 | 10 | -4396.23 | 2.1818 | 125.84 | 74.47 | 34.13287 |
| 3 | 10 | -4344.86 | 4.5417 | 51.37 | 62.39 | 13.737123 |
| 4 | 10 | -4355.88 | 19.242 | -11.02 | 43.18 | 2.244048 |
| 5 | 10 | -4410.08 | 64.3602 | -54.2 | 36.37 | 0.565101 |
| 6 | 10 | -4500.65 | 89.2156 | -90.57 | 85.64 | 0.959922 |
| 7 | 10 | -4676.86 | 215.5926 | -176.21 | 24.94 | 0.115681 |
| 8 | 10 | -4828.13 | 247.8569 | -151.27 | 162.57 | 0.655903 |
| 9 | 10 | -4816.83 | 217.635 | 11.3 | 97.61 | 0.448503 |
| 10 | 10 | -4707.92 | 277.7238 | 108.91 |  |  |


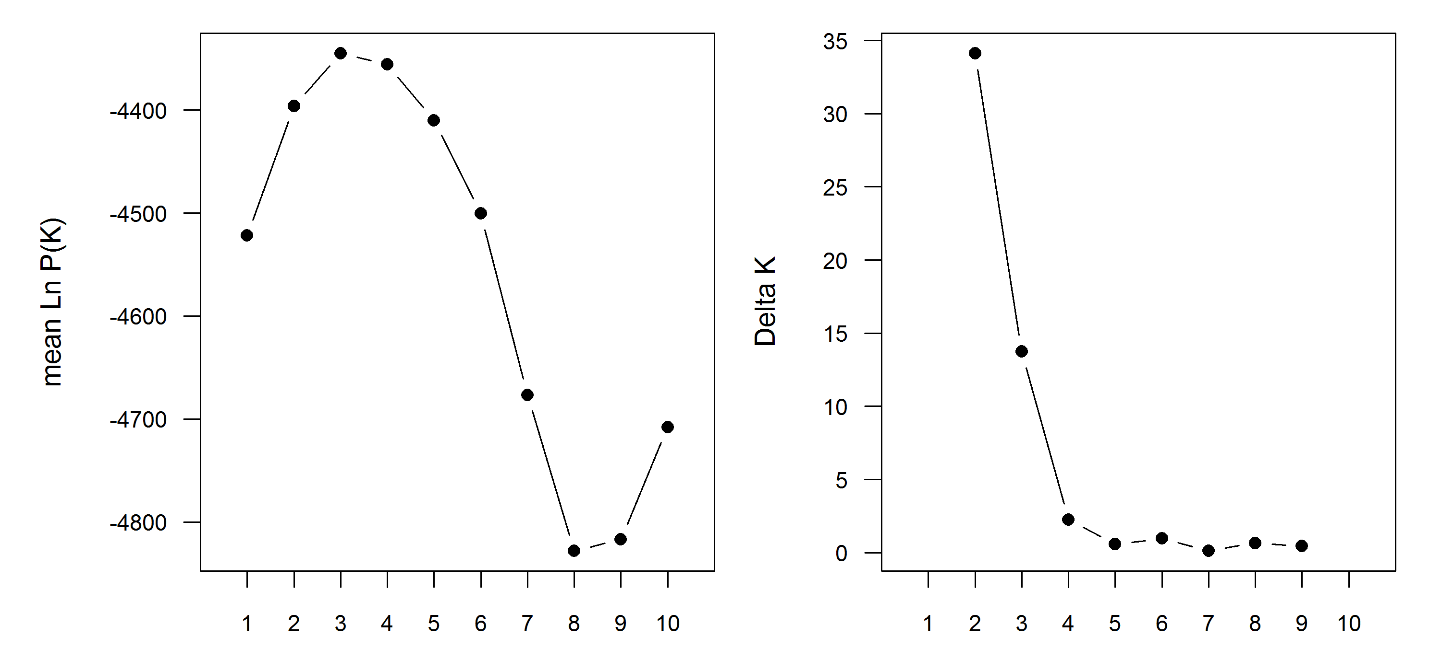


**Figure B.** Plot of mean Ln P(K) showing support for K = 3 for the subset of pika genotypes assigned to the northern cluster in the first Structure analysis. The Delta K plot is ambiguous, but suggests 1 to 3 genetic clusters.


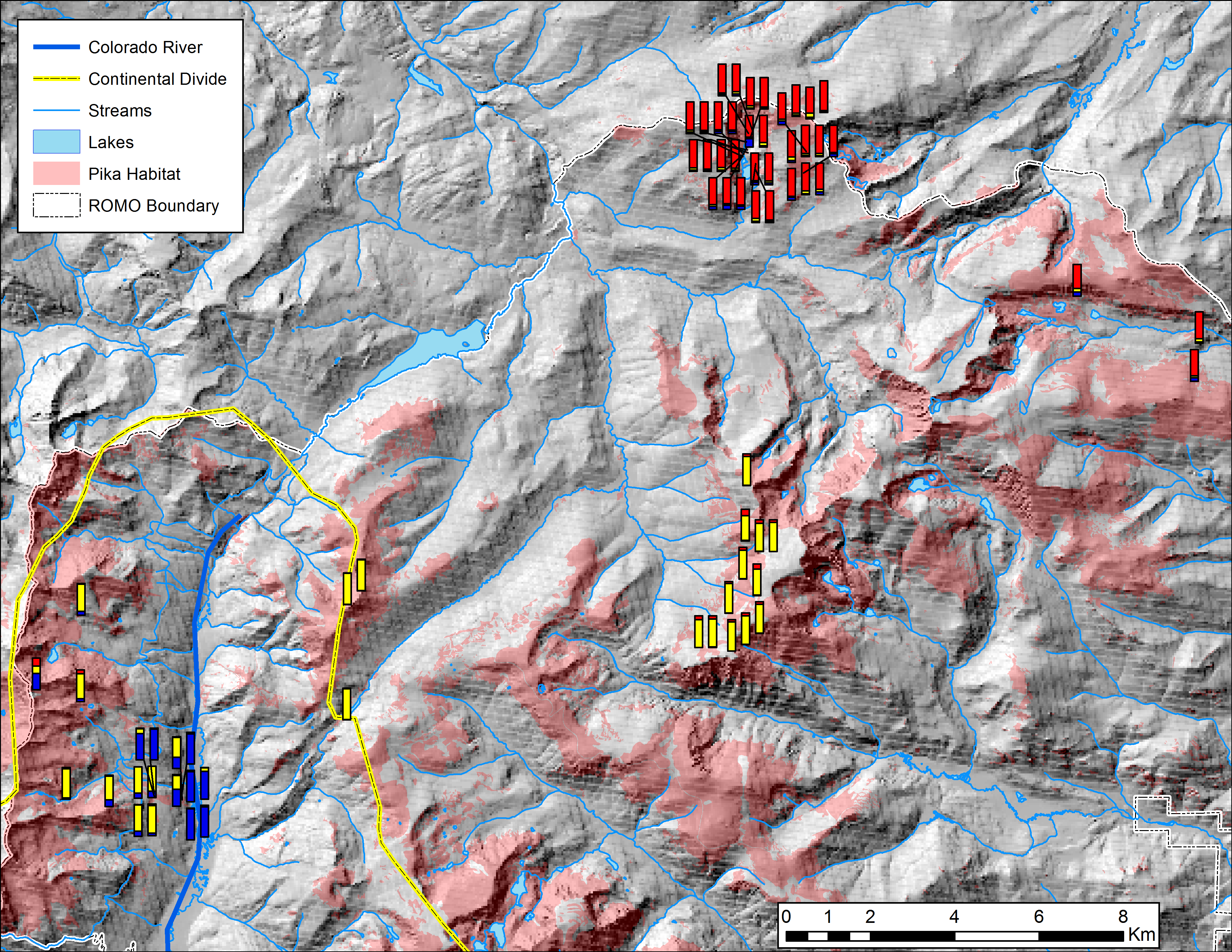


**Figure C.** Individuals assigned to the northern cluster in the first Structure analysis are shown as bar plots representing proportion of assignment (*Q* values) from the subsequent Structure analysis for K = 3. Here, individuals cluster predominantly into northern and southern groups, with the blue cluster found almost exclusively to the west of the Colorado River.


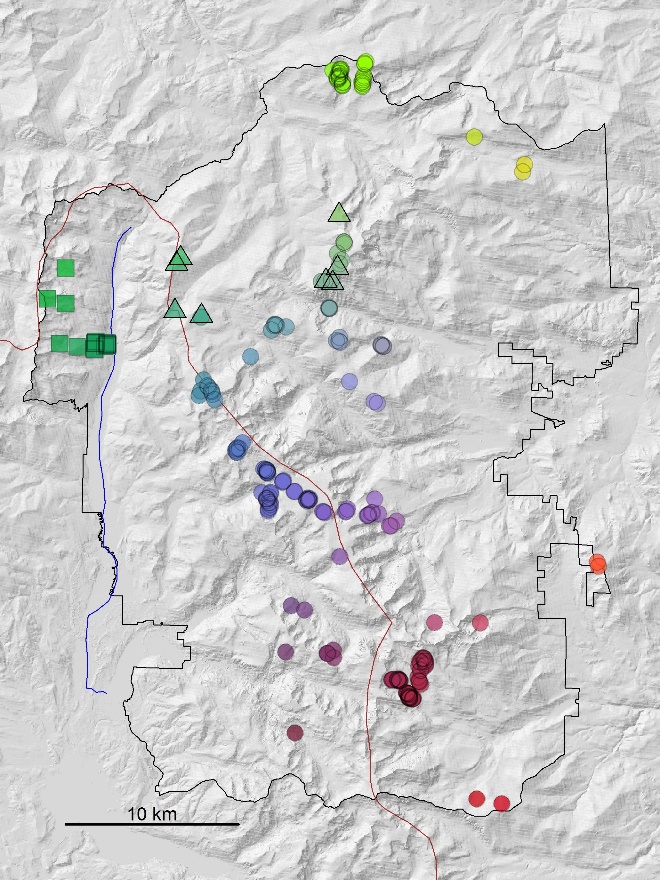


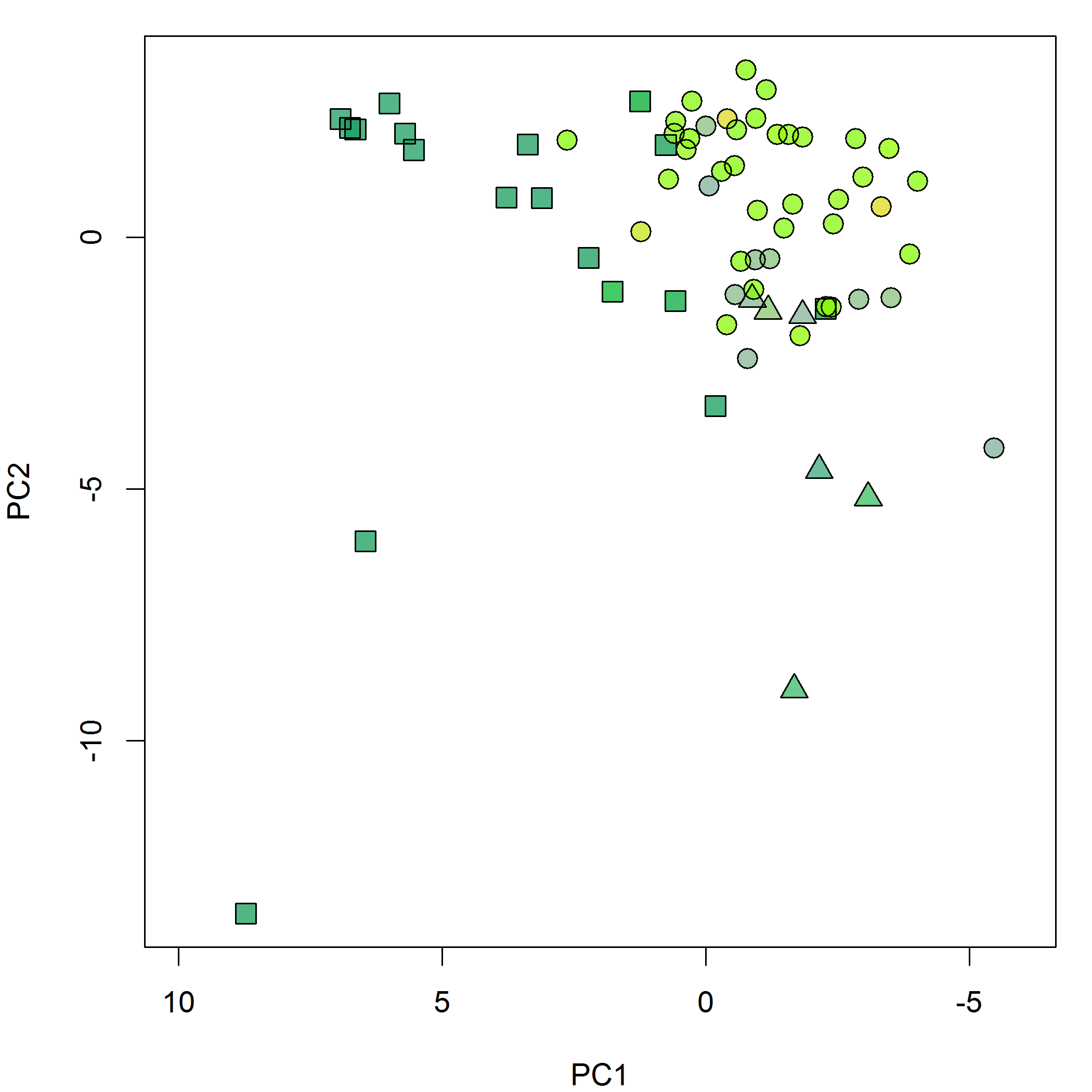


**Figure D.** Principal components analysis for the subset of pika genotypes assigned to the northern cluster with admixture proportion *Q* ≥ 0.6 in the first Structure analysis (Left). Colors in the PCA correspond to geographic localities shown on the map (Right). Triangles are individuals with 0.2 < *Q* < 0.8.

**Table C.** Summary of each of ten Structure runs for K = 1 to 10 for the subset of pika genotypes assigned to the southern cluster with admixture proportion *Q* ≥ 0.6 in the first Structure analysis. There is some support for K = 2 and K = 6.

| K | Reps | Mean LnP(K) | Stdev LnP(K) | Ln'(K) | \|Ln''(K)\| | Delta K |
| --- | --- | --- | --- | --- | --- | --- |
| 1 | 10 | -11284.9 | 1.2272 | NA | NA | NA |
| 2 | 10 | -11191.4727 | 11.5515 | 93.427273 | 63.754545 | 5.519177 |
| 3 | 10 | -11161.8 | 48.8146 | 29.672727 | 2.1 | 0.04302 |
| 4 | 10 | -11130.0273 | 54.8771 | 31.772727 | 4.081818 | 0.074381 |
| 5 | 10 | -11102.3364 | 82.1919 | 27.690909 | 16.8 | 0.2044 |
| 6 | 10 | -11091.4455 | 54.2991 | 10.890909 | 85.818182 | 1.580472 |
| 7 | 10 | -11166.3727 | 97.8816 | -74.927273 | 94.154545 | 0.961922 |
| 8 | 10 | -11147.1455 | 99.7682 | 19.227273 | 78.3 | 0.784819 |
| 9 | 10 | -11206.2182 | 170.2475 | -59.072727 | 40.745455 | 0.239331 |
| 10 | 10 | -11224.5455 | 84.0897 | -18.327273 | NA | NA |


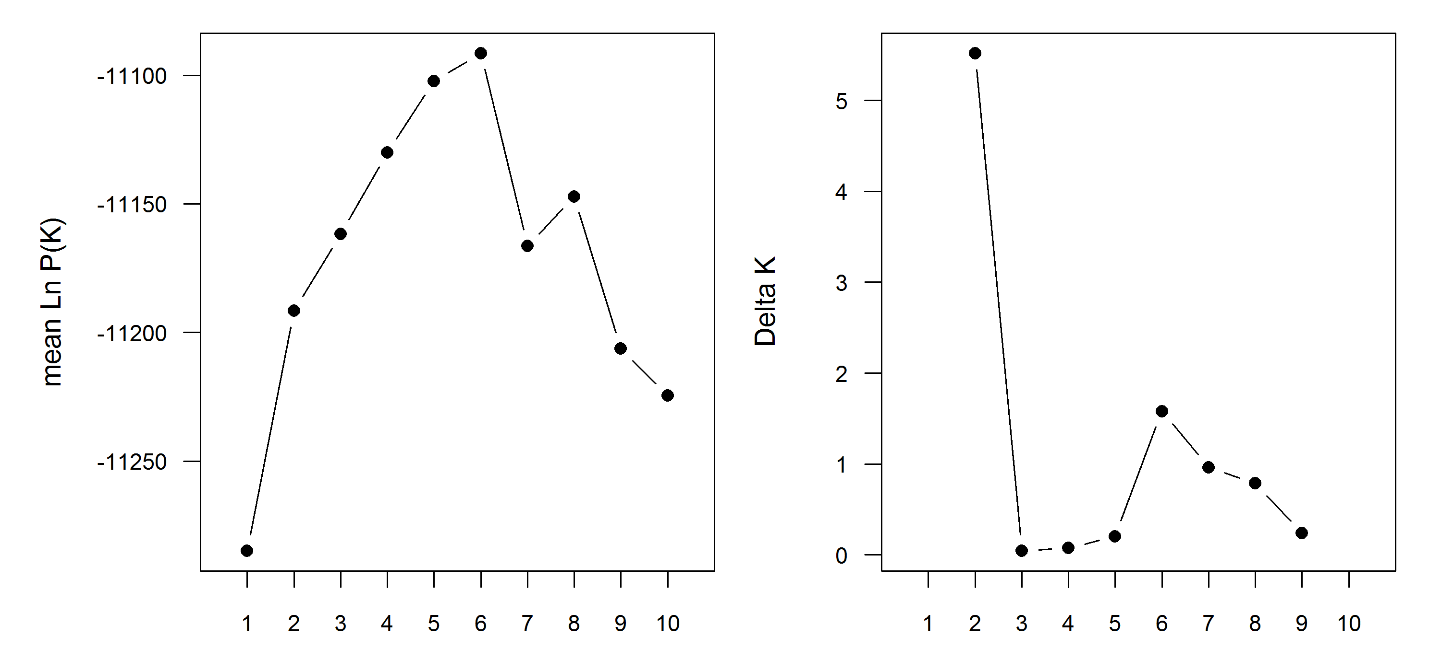


**Figure E.** Plot of mean Ln P(K) and Delta K showing support for K = 6 for the subset of pika genotypes assigned to the southern cluster in the first Structure analysis.


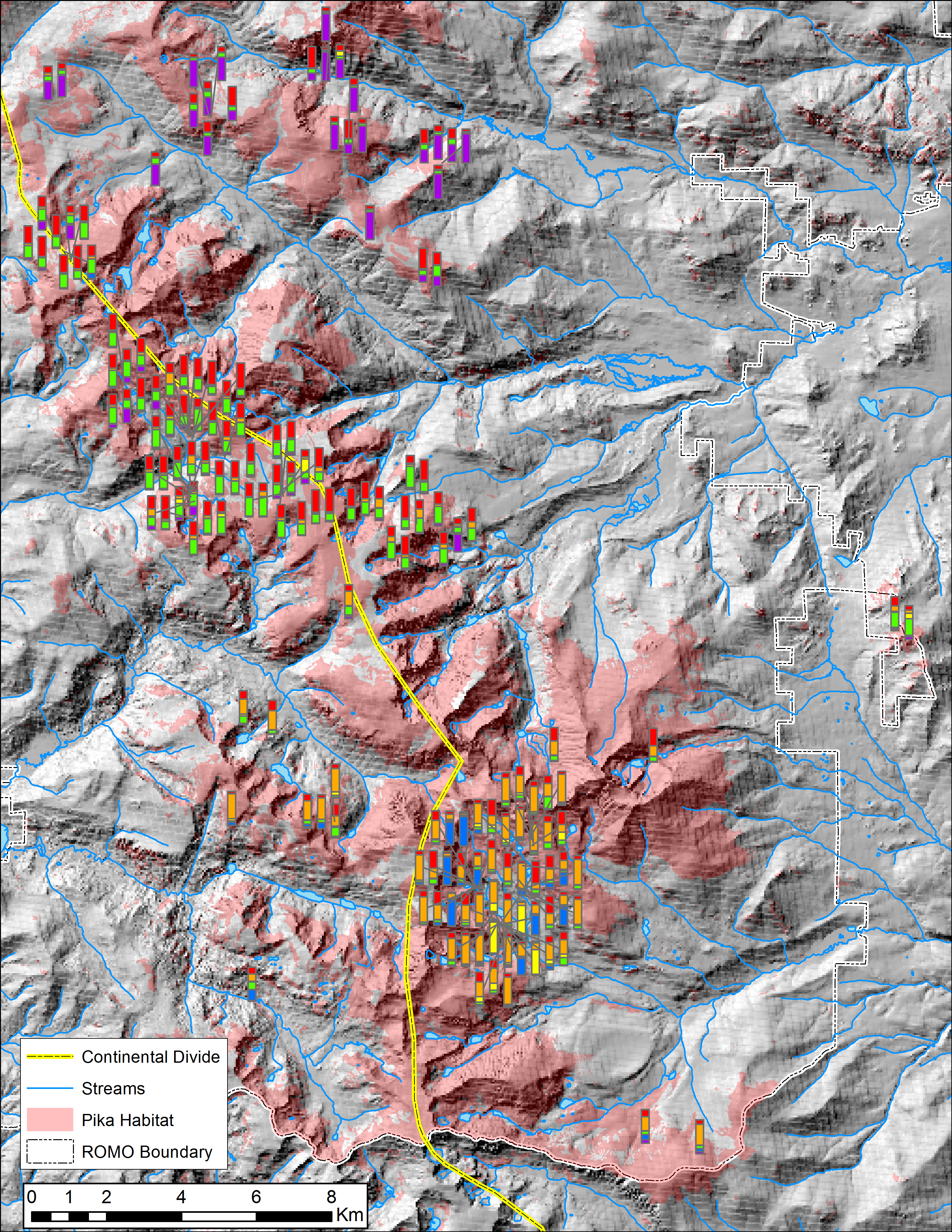


**Figure F.** Individuals assigned to the southern cluster in the first Structure analysis are shown as bar plots representing proportion of assignment (*Q* values) from the subsequent Structure analysis for K = 6. Here, individuals cluster predominantly into northern, central, and southern groups.


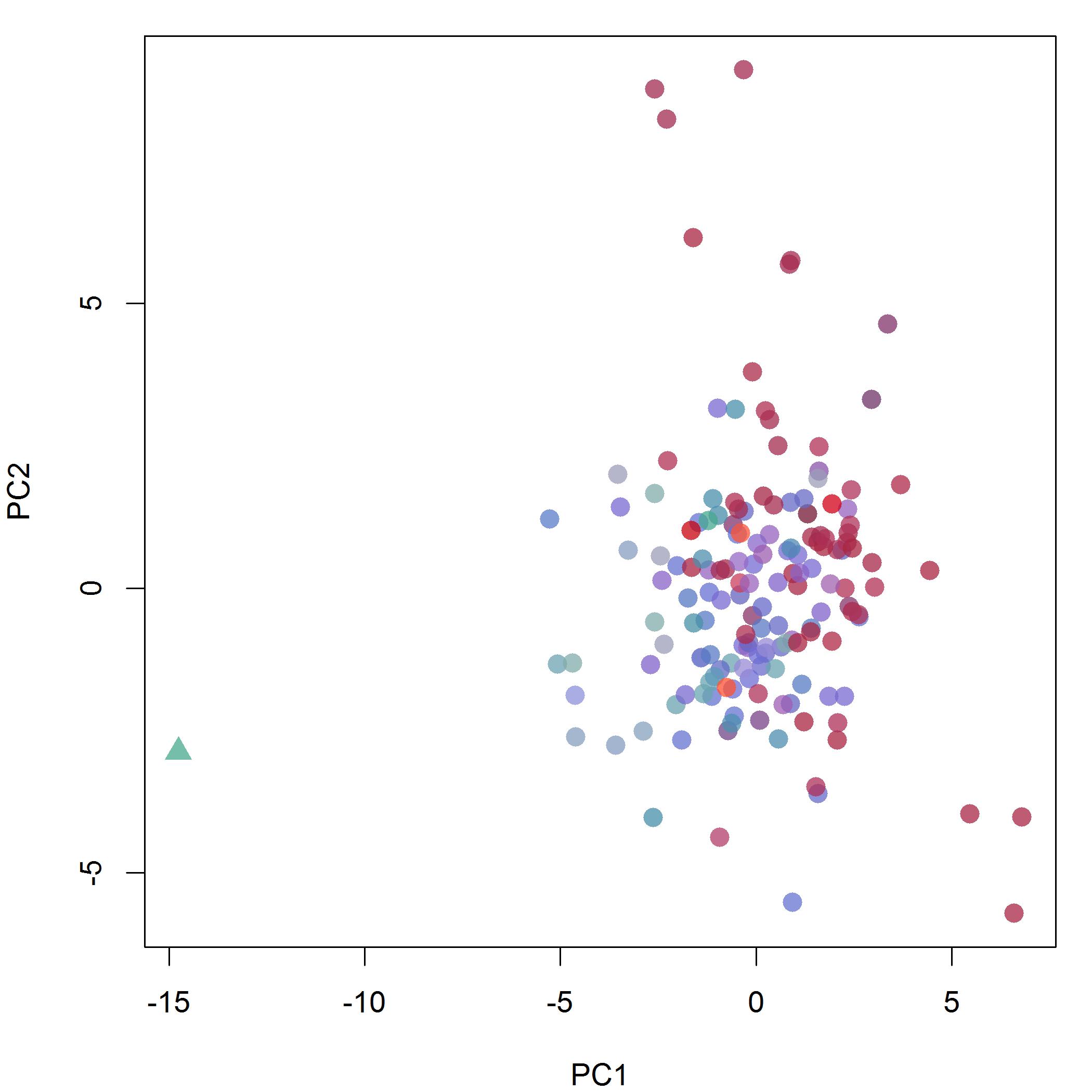

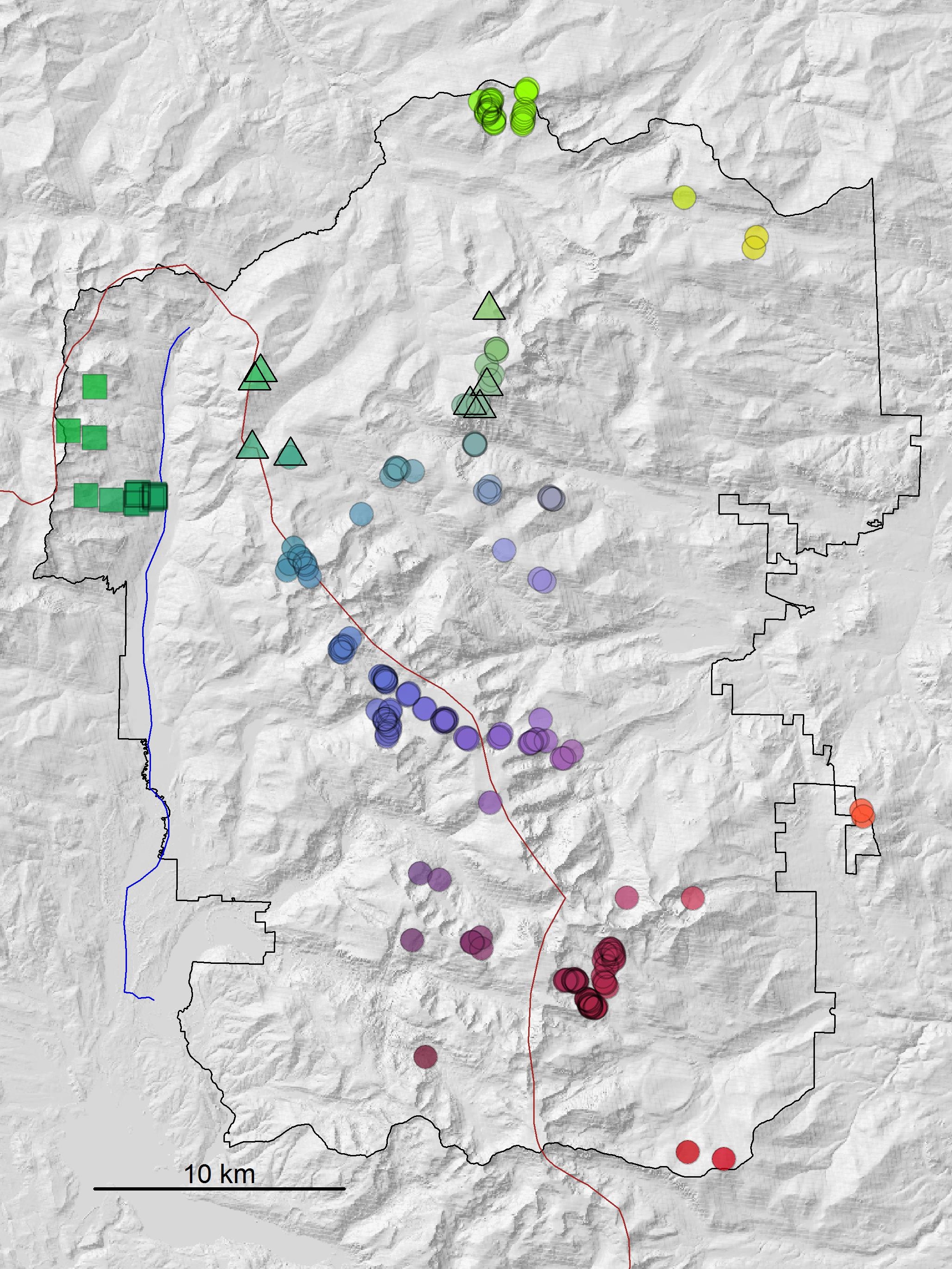


**Figure G.** Principal components analysis for the subset of pika genotypes assigned to the southern cluster with admixture proportion *Q* ≥ 0.6 in the first Structure analysis (Left). Colors in the PCA correspond to geographic localities shown on the map (Right). Triangles are individuals with 0.2 ≤ *Q* < 0.8. The first principal component reflects a latitudinal gradient similar to the pattern in Figure E.

**Table E.** Parameter estimates for the maximum likelihood clines estimated from STRUCTURE Admixture proportion *Q* using the program *HZAR.* Allele frequence parameters reflect whether frequencies were fixed at 0 and 1 (none), reflect the observed min and max (fixed), or are estimated for the ends of the tails (free). Two log-likelihood support limits for cline width are presented in parentheses. The best supported model is presented in Figure H.

| Input | Pmin | Pmax | Tails | Cline Center | Cline Width | log-likelihood | AICc |
| --- | --- | --- | --- | --- | --- | --- | --- |
| fixed | 0.00 | 1.00 | none | 27724.44 | 8566.96 (6500.37 - 11261.87) | -49.90 | 103.83 |
| none | 0.00 | 1.00 | none | 27740.54 | 8723.71 (6734.63 - 11391.56) | -50.04 | 104.10 |
| none | 0.00 | 1.00 | left | 27813.59 | 8422.81 (6887.58 - 11131.18) | -49.71 | 107.50 |
| free | 0.00 | 1.00 | none | 27718.12 | 8538.45 (6378.74 - 11009.57) | -49.80 | 107.70 |
| fixed | 0.00 | 1.00 | mirror | 27622.08 | 8535.51 (6528.72 - 11180.78) | -49.83 | 107.74 |
| none | 0.00 | 1.00 | mirror | 27590.49 | 8573.77 (6802.35 - 11237.09) | -49.85 | 107.78 |
| fixed | 0.00 | 1.00 | right | 27722.10 | 8585.37 (6491.77 - 11252.46) | -49.90 | 107.89 |
| fixed | 0.00 | 1.00 | left | 27732.27 | 8563.85 (6496.64 - 11243.84) | -49.90 | 107.89 |
| none | 0.00 | 1.00 | right | 27733.76 | 8723.58 (6090.76 - 11380.45) | -50.04 | 108.17 |
| none | 0.00 | 1.00 | both | 26614.99 | 5784.88 (5459.87 - 10962.49) | -49.51 | 111.20 |
| fixed | 0.00 | 1.00 | both | 26529.73 | 5540.75 (5540.75 - 11081.02) | -49.66 | 111.50 |
| free | 0.00 | 1.00 | right | 27687.76 | 8505.11 (6380.73 - 10952.13) | -49.83 | 111.85 |
| free | 0.00 | 1.00 | left | 27913.51 | 8802.66 (6315.26 - 11042.96) | -49.93 | 112.05 |
| free | 0.01 | 1.00 | both | 23778.88 | 22479.44 (21519.45 - 24071.54) | -120.69 | 257.69 |
| free | 0.01 | 0.86 | mirror | 20629.39 | 0.39 (0.39 - 140.23) | -129.47 | 271.12 |
| null |  |  |  |  |  | -242.66 | 487.33 |


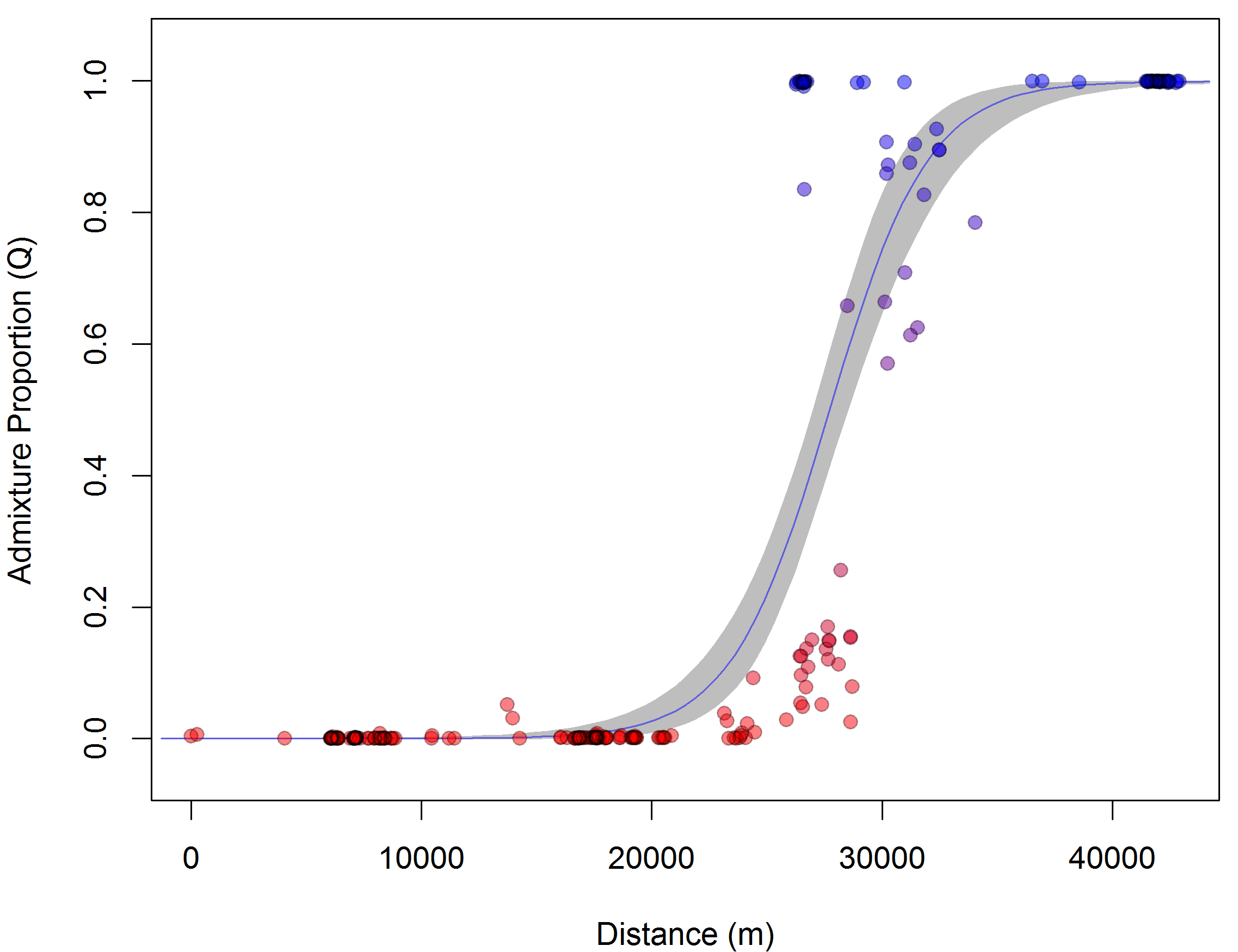


**Figure H.** Proportion of the genotype originating from the northern genetic cluster (STRUCTURE Admixture proportion *Q*) plotted against latitudinal distance from the southernmost individual with the maximum likelihood cline (blue line) and 95% credible cline region (grey shading) from the best cline model (Table E).


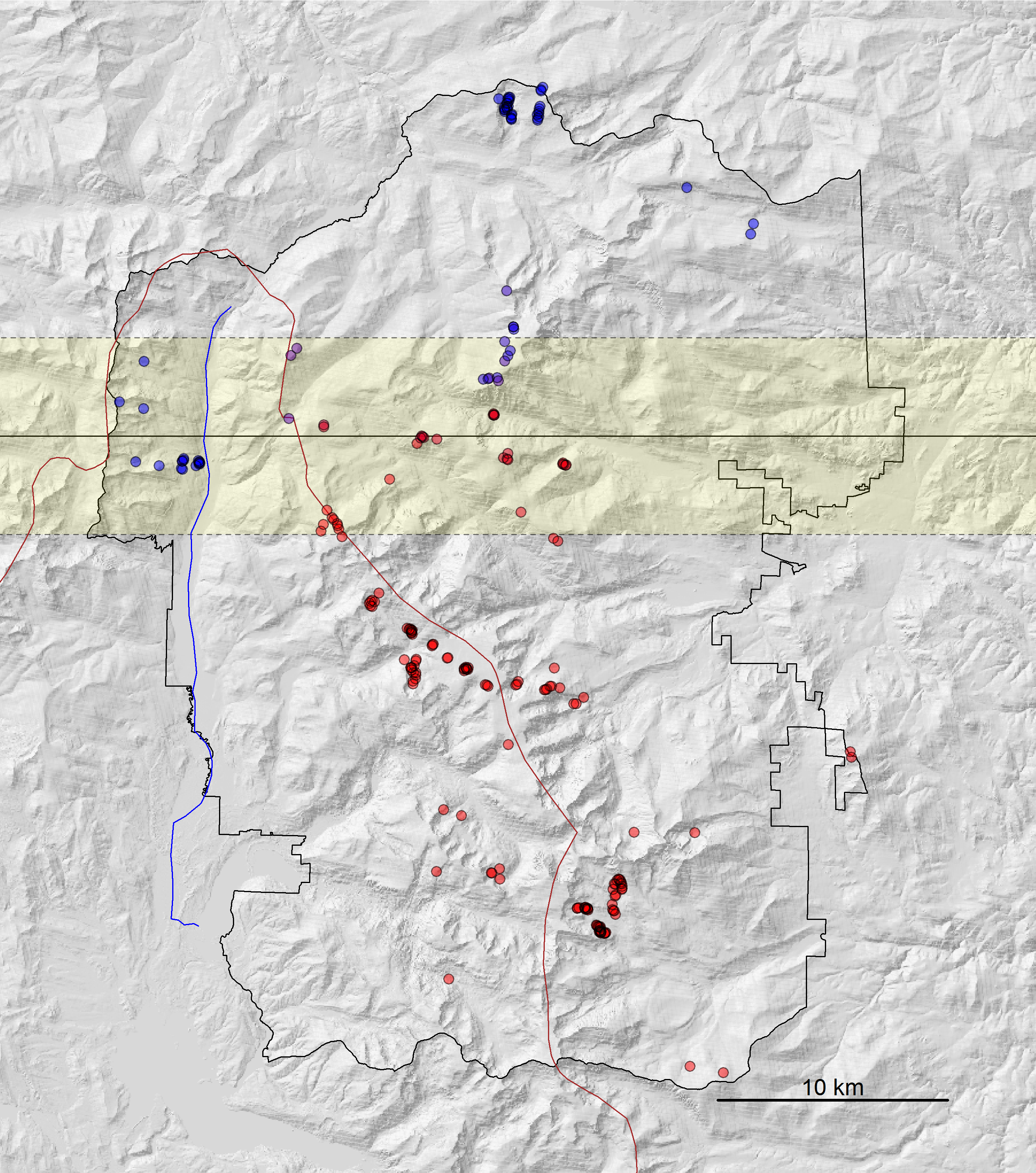


**Figure I.** Map of ROMO showing individual samples (colored according to Structure assignment probability, *Q*), the estimated location of the cline center (horizontal black line) and cline width (yellow shaded area), the continental divide (brown), and the Colorado River (blue line).


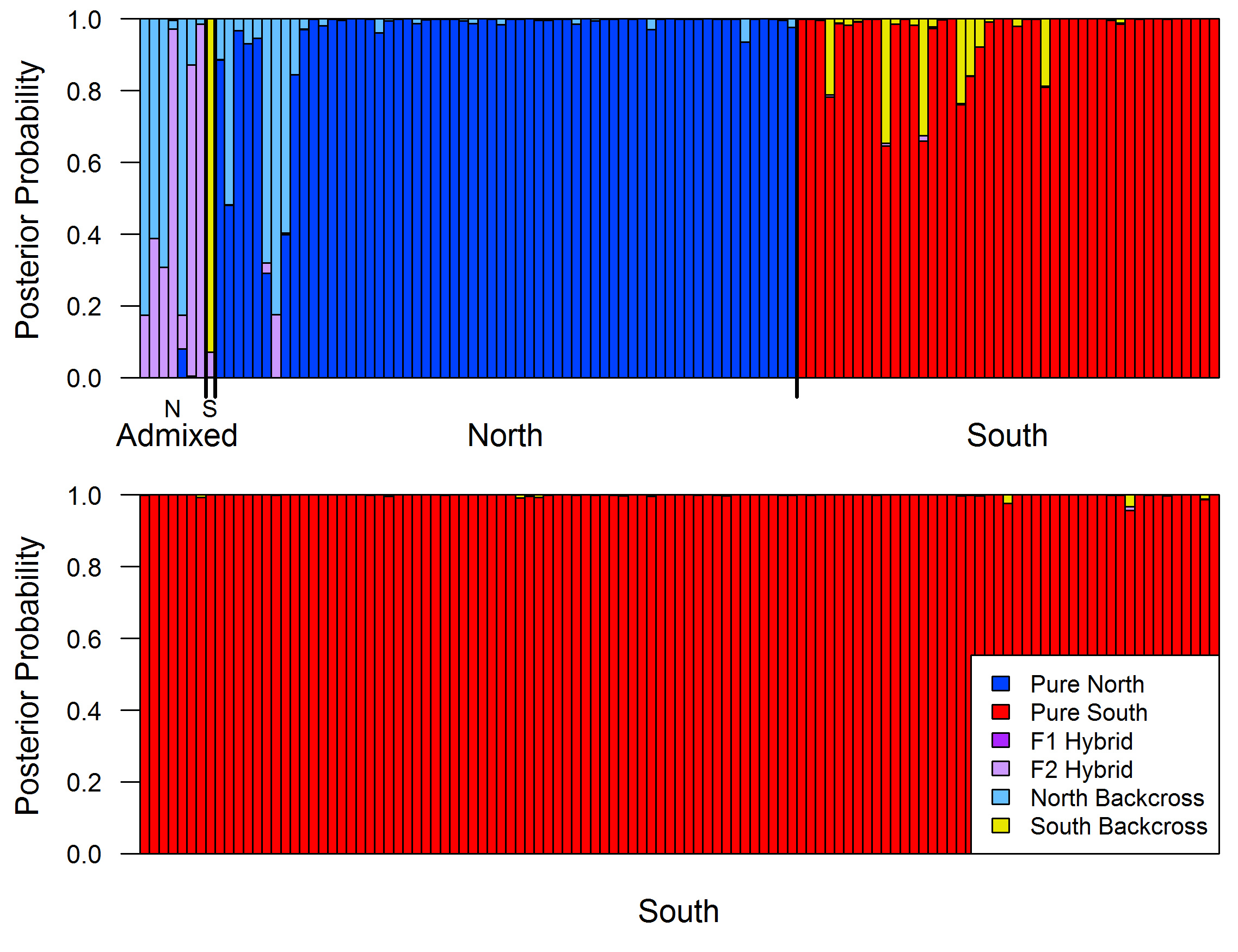


**Figure J.** Posterior probabilities of genotype frequency categories from the newhybrids analysis. X-axis labels reflect the presumed population of origin based on the initial Structure analysis with K = 2. Admixed individuals from the geographic north were either F2 hybrids or north backcrosses while the admixed individual from the geographic south was a south backcross.
